# μ_3_-Acetato-μ_2_-acetato-(di­methyl­form­amide)­penta­kis­(μ-*N*,2-dioxido­benzene-1-carboximidato)penta­kis­(1-methyl-1*H*-imidazole)­penta­manganese(III)manganese(II)–diethyl ether–di­methyl­formamide–methanol–water (1/1/1/1/0.49)

**DOI:** 10.1107/S1600536813015857

**Published:** 2013-06-15

**Authors:** Benjamin R. Tigyer, Matthias Zeller, Curtis M. Zaleski

**Affiliations:** a Department of Chemistry, Shippensburg University, 1871 Old Main Dr., Shippensburg, PA 17257, USA; b Department of Chemistry, Youngstown State University, 1 University Plaza, Youngstown, OH 44555, USA

## Abstract

The title compound, [Mn_6_(C_7_H_4_NO_3_)_5_(CH_3_CO_2_)_2_(C_4_H_6_N_2_)_4.62_(C_3_H_7_NO)_1.38_]·(C_2_H_5_)_2_O·C_3_H_7_NO·CH_3_OH·0.49H_2_O or Mn^II^(OAc)_2_[15-MC_Mn(III)N(shi)_-5](Me—Im)_4.62_(DMF)_1.38_·diethyl ether·DMF·MeOH·0.49H_2_O (where MC is metallacrown, ^−^OAc is acetate, shi^3−^ is salicyl­hydroximate, Me—Im is 1-methyl­imidazole, DMF is *N*,*N*-di­methyl­formamide, and MeOH is methanol), is comprised of five Mn^III^ ions in the metallacrown ring and an Mn^II^ ion which is encapsulated in the central cavity. Four of the ring Mn^III^ ions are six-coordinate with distorted octa­hedral geometries. Two of these Mn^III^ ions have a planar configuration, while the other two Mn^III^ have Λ absolute stereoconfiguration. The fifth Mn^III^ is five-coordinated with distorted square-pyramidal geometry. Four of the ring Mn^III^ ions each bind one 1-methyl­imidazole, while the final ring Mn^III^ ion binds a DMF solvent mol­ecule in an axial position and located in a *trans* position is either a Me—Im or a DMF mol­ecule. The occupancy ratio of Me—Im to DMF is 0.62 (2) to 0.38 (2). The central Mn^II^ is seven-coordinate with a geometry best described as distorted face-capped trigonal–prismatic. DMF, diethyl ether, MeOH, and water mol­ecules are located in the inter­stitial voids between the metallacrown mol­ecules. The methanol mol­ecule is positionally disordered [0.51 (1):0.49 (1)] and associated with a partially occupied water mol­ecule [0.49 (1)]. This disorder is also associated with the positional disorder of the diethyl ether mol­ecule [0.51 (1):0.49 (1)].

## Related literature

For a general review of metallacrowns, see: Mezei *et al.* (2007 ▶). For related manganese and vanadium metallacrown structures, see: Lah & Pecoraro (1989 ▶) and Pecoraro (1989 ▶), respectively. For related Mn(II)[15-MC_Mn(III)N(shi)_-5)] structures and synthetic procedures, see: Kessissoglou *et al.* (1994 ▶), Dendrinou-Samara *et al.* (2001 ▶, 2002 ▶, 2005 ▶); Emerich *et al.* (2010 ▶); Tigyer *et al.* (2011 ▶, 2012 ▶). For an explanation on how to calculate the *s/h* ratio, see: Stiefel & Brown (1972 ▶). For an explanation on how to calculate bond-valence-sum values, see: Liu & Thorp (1993 ▶). For an explanation on how to calculate the τ asymmetry parameter, see: Addison *et al.* (1984 ▶). For *CELL_NOW* software, see: Sheldrick (2008*b* ▶). 
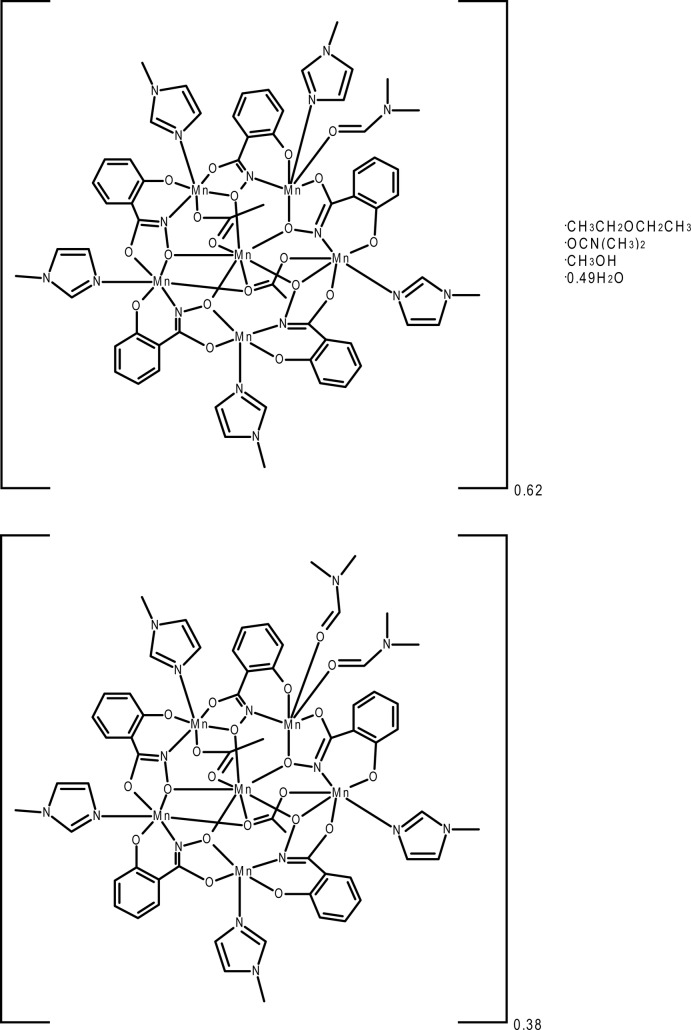


## Experimental

### 

#### Crystal data

[Mn_6_(C_7_H_4_NO_3_)_5_(C_2_H_3_O_2_)_2_(C_4_H_6_N_2_)_4.62_(C_3_H_7_NO)_1.38_]·C_4_H_10_O·C_3_H_7_NO·CH_4_O·0.49H_2_O
*M* *_r_* = 1866.61
Triclinic, 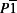

*a* = 12.4181 (8) Å
*b* = 17.0108 (11) Å
*c* = 20.6627 (13) Å
α = 102.166 (4)°
β = 96.726 (4)°
γ = 107.496 (4)°
*V* = 3992.4 (5) Å^3^
*Z* = 2
Mo *K*α radiation
μ = 1.01 mm^−1^
*T* = 100 K
0.30 × 0.23 × 0.15 mm

#### Data collection

Bruker SMART APEX CCD diffractometer
Absorption correction: multi-scan (*TWINABS*; Sheldrick, 2009 ▶) *T*_min_ = 0.544, *T*_max_ = 0.747
56608 measured reflections
18890 independent reflections
13018 reflections with *I* > 2σ(*I*)
*R*_int_ = 0.134

#### Refinement

*R*[*F* ^2^ > 2σ(*F* ^2^)] = 0.087
*wR*(*F* ^2^) = 0.232
*S* = 1.04
18890 reflections
1146 parameters
93 restraints
H atoms treated by a mixture of independent and constrained refinement
Δρ_max_ = 1.07 e Å^−3^
Δρ_min_ = −1.08 e Å^−3^

### 

Data collection: *APEX2* (Bruker, 2012 ▶); cell refinement: *SAINT* (Bruker, 2012 ▶); data reduction: *SAINT*; program(s) used to solve structure: *SHELXS97* (Sheldrick, 2008*a* ▶); program(s) used to refine structure: *SHELXL2012* (Sheldrick, 2008*a* ▶) and *SHELXLE Rev600* (Hübschle *et al.*, 2011 ▶); molecular graphics: *Mercury* (Macrae *et al.*, 2006 ▶); software used to prepare material for publication: *publCIF* (Westrip, 2010 ▶).

## Supplementary Material

Crystal structure: contains datablock(s) I, global. DOI: 10.1107/S1600536813015857/jj2164sup1.cif

Structure factors: contains datablock(s) I. DOI: 10.1107/S1600536813015857/jj2164Isup2.hkl

Additional supplementary materials:  crystallographic information; 3D view; checkCIF report

## Figures and Tables

**Fig. 1.**
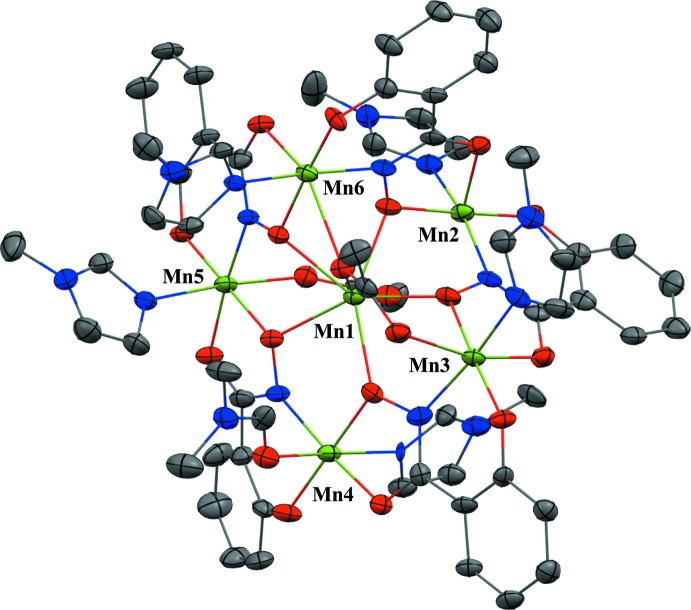
Single-crystal X-ray structure of Mn(II)(OAc)2[15-MCMn(III)N(shi)-5](Me—Im)4.62(DMF)1.38.diethyl ether.DMF.MeOH.0.49H2O (1). The thermal ellipsoid plot of 1 is at a 50% probability level. For Mn4 only the 1-methylimidazole is shown bound to the MnIII, since 1-methylimidazole possess a higher occupancy ratio compared to the coordinated DMF (0.62 (2):0.38 (2)). Hydrogen atoms and the lattice solvent molecules have been omitted for clarity. Color scheme for all figures: green - MnII and MnIII, red - oxygen, blue - nitrogen, and gray - carbon.

**Fig. 2.**
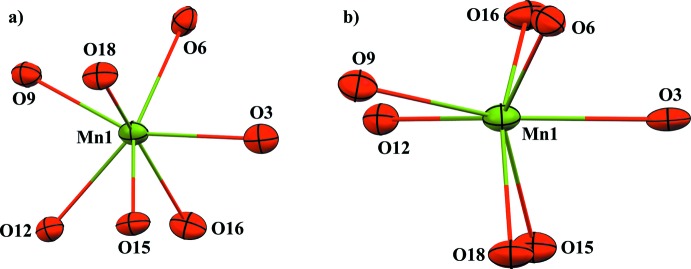
Side (a) and top (b) views of the first coordination sphere about Mn1 (2+ oxidation state) of 1. The thermal ellipsoid plots are at a 50% probability level.

**Fig. 3.**
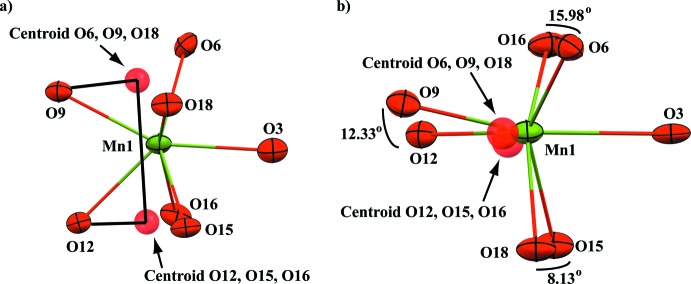
Side (a) and top (b) views of the first coordination sphere about Mn1 of 1 demonstrating how the azimuthal anlge (Φ) was defined and calculated using the program Mercury (Macrae et al., 2006). The thermal ellipsoid plots are at a 50% probability level.

**Fig. 4.**
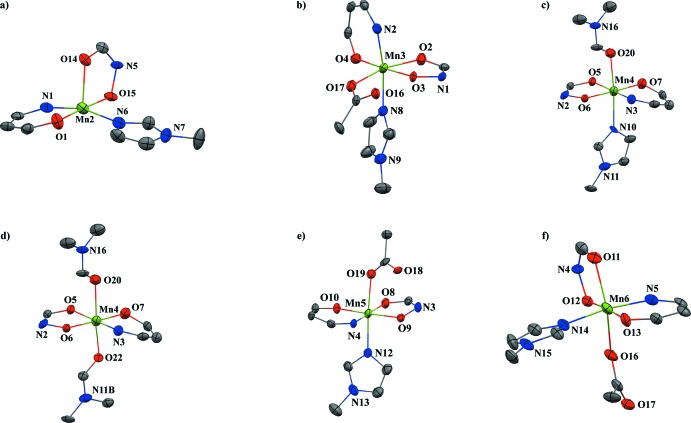
First coordination sphere about each MnIII ion of 1. a) Mn2 with distorted square pyramidal geometry b) Mn3 with Λ configuration c) Mn4 with planar configuration and 1-methylimidazole bound (0.62 (2) occupancy) d) Mn4 with planar configuration and DMF bound (0.38 (2) occupancy) e) Mn5 with planar configuration and f) Mn6 with Λ configuration. The thermal ellipsoid plots are at a 50% probability level. Hydrogen atoms have been omitted for clarity.

## supplementary crystallographic information

### Comment

Metallacrowns were first recognized in 1989 by Pecoraro, and since then they have proven to be a versatile class of inorganic compounds (Pecoraro, 1989; Mezei *et al.*, 2007). They have served as building blocks for 1-, 2-, and three-dimensional solids, displayed interesting relaxivity behavior, and served as selective-anion hosts (Mezei *et al.*, 2007). In addition, the manganese-based 15-MC-5 compounds have shown enhanced antimicrobial properties compared to simple Mn-herbicide compounds (Kessissoglou *et al.*, 1994; Dendrinou-Samara *et al.*, 2001, 2002, 2005). These initial manganese-based 15-MC-5 compounds where made using pyridine to complete the coordination of the ring Mn^III^ ions. However, recently it has been shown that imidazole and its derivatives can also be readily used to produce a manganese 15-MC-5 compound (Emerich *et al.*, 2010; Tigyer *et al.* 2011, 2012).

Herein we report the synthesis, IR data, and single-crystal X-ray structure of the title compound [Mn_6_(C_7_H_4_NO_3_)_5_(C_2_H_3_O_2_)_2_(C_4_H_6_N_2_)_4.62_(C_3_H_7_NO)_1.38_].(C_2_H_5_)_2_O.C_3_H_7_NO.CH_3_OH.0.49H_2_O, 1, abbreviated as Mn(II)(OAc)_2_[15-MC_Mn(III)_*_N_*_(shi)_-5](Me—Im)_4.62_(DMF)_1.38_.diethyl ether.DMF.MeOH.0.49H_2_O (where MC is metallacrown, ^-^OAc is acetate, shi^3-^ is salicylhydroximate, Me—Im is 1-methylimidazole, DMF is *N*,*N*-dimethylformamide, and MeOH is methanol). The molecule is nonplanar, which is typical of manganese-based 15-MC-5 complexes (Fig. 1). The MC framework of the molecule is comprised five shi^3-^ ligands and five Mn^III^ ions, which combine to form a -[Mn^III^—N—O]_5_– repeat unit. A Mn^II^ ion is captured in the central cavity of the MC and the Mn^II^ ion is tethered to the MC ring *via* two acetate ligands. Charge neutrality in the molecule is maintained by the five Mn^III^ and one Mn^II^ cations and five shi^3-^ and two acetate ligands.

Mn1 is located in the central cavity and is seven-coordinate with distorted face-capped trigonal prismatic geometry (Fig. 2). The geometry assignment is supported by both the calculated azimuthal angle (Φ) and the *s/h* ratio (Stiefel & Brown, 1972). These parameters can be used to distinguish an ideal trigonal prism and octahedron. In an ideal trigonal prism the angle between the atoms on opposite triangular faces is Φ = 0°, and the *s/h* ratio is 1.00. In an ideal octahedron the azimuthal angle equals 60°, and the *s/h* ratio is 1.22. To calculate these parameters the centroids of opposite triangular faces made by the donor oxygen atoms (O6, O9, and O18; O12, O15, and O16) were defined using the program *Mercury* (Fig. 3; Macrae *et al.*, 2006). The azimuthal angles were measured between atoms on opposite faces through the centroids. To calculate the *s/h* ratio, the distance between the centroids was defined as *h*, and the distances between atoms on the same triangular face were defined as *s*. For Mn1 the Φ angles are 8.13°, 12.33°, and 15.98°, and the estimated average *s/h* ratio is 1.01±0.11. Thus, both the Φ angle and *s/h* parameters support a distorted faced-capped trigonal prismatic geometry. Mn1 is assigned a 2+ oxidation state which is supported by an average bond distance of 2.24 Å and a Bond Valence Sum (BVS) calculation of 1.92 (Liu & Thorp, 1993).

The ring Mn2 - Mn6 ions have various coordination modes and configurations (Fig. 4). Mn2 is five-coordinate (Fig. 4a) with distorted square pyramidal geometry. To evaluate the geometry about Mn2 the τ parameter was calculated (Addison *et al.*, 1984). For an ideal square pyramidal geometry τ = 0, while for an ideal trigonal bipyramidal geometry τ = 1. For Mn2 τ is 0.21. Mn3 - Mn 6 are six-coordinate with distorted octahedral geometry. In addition, the coordination about these Mn can be described by their configurations. Mn3 (Fig. 4 b) and Mn6 (Fig. 4 e) have a propeller configuration of two chelate rings of different shi^3-^ ligands with Λ absolute stereochemistry. Mn4 (Fig. 4c) and Mn5 (Fig. 4 d) adopt a planar (P) configuration, where two chelate rings of different shi^3-^ ligands are located *trans* to each other. In addition, Mn2, Mn3, Mn5, and Mn6 each bind one 1-methylimidazole ligand, which is directed to the periphery of the metallacrown. Mn4 binds one DMF molecule in an axial position and located in a *trans* position is either a 1-methylimidazole or a DMF. The occupancy ratio of 1-methylimidazole to DMF is 0.62 (2) to 0.38 (2). Mn2 - Mn6 are assigned a 3+ oxidation state, which is supported by the average bond distances and BVS calculations. The average Mn-N/O bond distances for Mn2, Mn3, Mn4, Mn5, and Mn6 are 1.98 Å, 2.04 Å, 2.06 Å, 2.04 Å, and 2.05 Å, respectively, and the BVS calculations are 2.99, 3.09, 3.04, 3.11, and 3.09, respectively. In addition, Mn3 - Mn6 possess a Jahn-Teller axis, which is typical for high spin *d*^4^ Mn^III^ ions.

Lastly DMF, diethyl ether, methanol, and water molecules are located in the interstitial voids between the metallacrown molecules. The methanol molecule is positional disordered [0.51 (1):0.49 (1)] and associated with a partially occupied water molecule [0.49 (1)]. This disorder is also associated with the positional disorder of the diethyl ether molecule [0.51 (1):0.49 (1)].

### Experimental

Manganese(II) acetate tetrahydrate (99+%) was purchased from Acros Organics. Salicylhydroxamic acid (H_3_shi, 99%) and 1-methylimidazole (99%) were purchased from Alfa Aesar. Methanol (HPLC grade) was purchased from Pharmco-AAPer. *N*,*N*-dimethylformamide (Certified ACS grade) was purchased from BDH chemicals. Absolute diethyl ether was purchased from EMD Chemicals. All reagents were used as received and without further purification.

The compound {Mn(II)(OAc)_2_[12-MC_Mn(III)N(shi)_-4](DMF)_6_.2DMF was prepared as previously reported (Lah & Pecoraro, 1989). Dark brown/black crystals were isolated and dried. Then the {Mn(II)(OAc)_2_[12-MC_Mn(III)N(shi)_-4](DMF)_6_.2DMF compound (0.1 mmol) was dissolved in 20 ml of a 75:25 solution of DMF and methanol resulting in a dark brown solution. Following 25 µ*L* of 1-methylimidazole was added and no change was observed. This solution was stirred for 5 minutes. Diffusion of diethyl ether into the solution at room temperature resulted in small black platelets suitable for X-ray analysis after 8 days. The percent yield was 6.8% based on {Mn(II)(OAc)_2_[12-MC_Mn(III)N(shi)_-4](DMF)_6_.2DMF.

Elemental analysis for the dried material (accounting for the loss of the diethyl ether lattice solvent) C_65.62_H_75.36_Mn_6_N_16.62_O_22.87_ [FW = 1792.43 g/mol] found % (calculated); C 44.30 (43.97); H 4.10 (4.24); N 13.34 (12.99).

### Refinement

The crystals under investigation were heavily intergrown and fragile and no single piece sufficiently large for XRD analysis could be obtained. Attempts to obtain single pieces from larger fragments through careful cutting were not successful due to the dark colour and fragility of the crystallites. Instead a sufficiently large fragment with three larger and a number of smaller moieties was chosen for analysis. The orientation matrices for the three largest moieties were identified using the program *CELL_NOW* (Sheldrick, 2008*b*) with the three components being not related by any obvious twin operations. The three components were integrated using *SAINT* (Bruker, 2012) resulting in the following statistics:

54454 data (16586 unique) involve domain 1 only, mean I/sigma 3.4

23789 data (11631 unique) involve domain 2 only, mean I/sigma 2.6

24251 data (11600 unique) involve domain 3 only, mean I/sigma 1.7

41797 data (20117 unique) involve 2 domains, mean I/sigma 3.0

25039 data (10789 unique) involve 3 domains, mean I/sigma 3.1

8 data (8 unique) involve 4 domains, mean I/sigma 1.2

The exact twin matrices identified by the integration program were found to be:

Matrix 1 → Matrix 2

0.96554 - 0.07897 0.03223

0.17039 1.03400 0.01296

-0.14548 - 0.01729 0.98326

Matrix 1 → Matrix 3

0.97038 - 0.06968 0.02764

0.16471 1.04753 0.07915

-0.18744 - 0.12396 0.95778

Matrix 2 → Matrix 3

1.00268 0.00911 - 0.00488

0.00169 1.01434 0.06707

-0.02835 - 0.10572 0.97641

The data were corrected for absorption using *TWINABS* (Sheldrick, 2009), and the structure was solved using direct methods with only the non-overlapping reflections of component 1. The structure was refined using the hklf 5 routine with all reflections of component 1 (including the overlapping ones) with a resolution better than 0.8 Å, resulting in BASF values of 0.301 (2) and 0.167 (2).

The total number of reflections given (_diffrn_reflns_number) is before the cutoff at 0.8 Å. The *R*_int_ value (_diffrn_reflns_av_R_equivalents) given is for these reflections and is based on agreement between observed single and composite intensities and those calculated from refined unique intensities and twin fractions before the cutoff at 0.8 Å (*TWINABS*).

One of the coordinated 1-methylimidazole ligands is partially replaced by a DMF molecule. Overlapping atoms were constrained to have identical ADPs and to be close to isotropic. The DMF molecule was restrained to have a geometry similar to that of another not disordered DMF molecule. The occupancy ratio refined to 0.61983 (2000) to 0.38017 (2000) in favor of the 1-methylimidazole molecule.

A methanol molecule is positional disordered with one of the molecules associated with a partially occupied water molecule. The disorder is associated with disorder of a diethyl ether molecule. Occupancy ratios of all three solvent molecules refined to essentially 1:1 (0.50926 (1100) to 0.49074 (1100)). The oxygen and carbon atoms of the methanol and water molecules were restrained to have similar ADPs (SIMU restraint in Shexltl).

Reflections 0 0 1 and 1 - 1 1 were obstructed by the beam stop and were omitted from the refinement.

### Crystal data

**Table:**

[Mn_6_(C_7_H_4_NO_3_)_5_(C_2_H_3_O_2_)_2_(C_4_H_6_N_2_)_4.62_(C_3_H_7_NO)_1.38_]·C_4_H_10_O·C_3_H_7_NO·CH_4_O·0.49H_2_O	*Z* = 2
*M**_r_* = 1866.61	*F*(000) = 1920.8
Triclinic, *P*1	*D*_x_ = 1.552 Mg m^−^^3^
Hall symbol: -P 1	Mo *K*α radiation, λ = 0.71073 Å
*a* = 12.4181 (8) Å	Cell parameters from 9924 reflections
*b* = 17.0108 (11) Å	θ = 2.2–27.6°
*c* = 20.6627 (13) Å	µ = 1.01 mm^−^^1^
α = 102.166 (4)°	*T* = 100 K
β = 96.726 (4)°	Plate, black
γ = 107.496 (4)°	0.30 × 0.23 × 0.15 mm
*V* = 3992.4 (5) Å^3^	

### Data collection

**Table:**

Bruker SMART APEX CCD diffractometer	13018 reflections with *I* > 2σ(*I*)
Radiation source: fine focus sealed tube	*R*_int_ = 0.134
ω and phi scans	θ_max_ = 26.4°, θ_min_ = 1.3°
Absorption correction: multi-scan (*TWINABS*; Sheldrick, 2009)	*h* = −15→15
*T*_min_ = 0.544, *T*_max_ = 0.747	*k* = −21→20
56608 measured reflections	*l* = 0→25
18890 independent reflections	

### Refinement

**Table:**

Refinement on *F*^2^	Primary atom site location: structure-invariant direct methods
Least-squares matrix: full	Secondary atom site location: difference Fourier map
*R*[*F*^2^ > 2σ(*F*^2^)] = 0.087	Hydrogen site location: difference Fourier map
*wR*(*F*^2^) = 0.232	H atoms treated by a mixture of independent and constrained refinement
*S* = 1.04	*w* = 1/[σ^2^(*F*_o_^2^) + (0.0741*P*)^2^ + 15.4132*P*] where *P* = (*F*_o_^2^ + 2*F*_c_^2^)/3
18890 reflections	(Δ/σ)_max_ < 0.001
1146 parameters	Δρ_max_ = 1.07 e Å^−^^3^
93 restraints	Δρ_min_ = −1.08 e Å^−^^3^

### Special details

**Table:**

Experimental. Di-µ-aceto-mono(dimethylformamide)pentakis(µ-*N*,2-dioxidobenzene-1-carboximidato)pentakis(1-methylimidazole)pentamanganese(III)manganese(II)–diethyl ether-dimethylformamide-methanol-water (1/1/1/0.49)FT–IR bands (KBr pellet, cm^-1^): 1669, 1653, 1598, 1570, 1500, 1437, 1421, 1389, 1320, 1258, 1243, 1146, 1102, 1033, 1025, 954, 926, 865, 753, 681, 669, 653, 616, 595, 577, 486, 469, 418, and 404.
Geometry. All e.s.d.'s (except the e.s.d. in the dihedral angle between two l.s. planes) are estimated using the full covariance matrix. The cell e.s.d.'s are taken into account individually in the estimation of e.s.d.'s in distances, angles, and torsion angles; correlations between e.s.d.'s in cell parameters are only used when they are defined by crystal symmetry. An approximate (isotropic) treatment of cell e.s.d.'s is used for estimating e.s.d.'s involving l.s. planes.
Refinement. Refined as a 3-component twin.

### Fractional atomic coordinates and isotropic or equivalent isotropic displacement parameters (Å^2^)

**Table:**

	*x*	*y*	*z*	*U*_iso_*/*U*_eq_	Occ. (<1)
Mn1	0.56026 (9)	0.25912 (7)	0.23833 (5)	0.0193 (3)	
Mn2	0.58160 (10)	0.32031 (7)	0.40739 (6)	0.0229 (3)	
Mn3	0.57536 (10)	0.06313 (7)	0.27180 (6)	0.0220 (3)	
Mn4	0.79276 (10)	0.23709 (8)	0.15153 (6)	0.0248 (3)	
Mn5	0.60155 (10)	0.43514 (8)	0.17163 (6)	0.0225 (3)	
Mn6	0.28559 (9)	0.25305 (8)	0.22500 (6)	0.0215 (3)	
O1	0.6762 (5)	0.3379 (4)	0.4890 (3)	0.0313 (14)	
O2	0.6924 (5)	0.1096 (3)	0.3655 (3)	0.0267 (13)	
O3	0.5555 (4)	0.1697 (3)	0.3059 (2)	0.0241 (12)	
O4	0.6034 (5)	−0.0397 (3)	0.2429 (3)	0.0268 (13)	
O5	0.8200 (4)	0.1295 (3)	0.1493 (3)	0.0231 (12)	
O6	0.6780 (4)	0.1918 (3)	0.2005 (2)	0.0227 (12)	
O7	0.9041 (5)	0.2796 (4)	0.1026 (3)	0.0319 (14)	
O8	0.7492 (5)	0.4685 (3)	0.1438 (3)	0.0276 (13)	
O9	0.6274 (4)	0.3267 (3)	0.1616 (2)	0.0223 (12)	
O10	0.5824 (5)	0.5419 (4)	0.1828 (3)	0.0273 (13)	
O11	0.2929 (5)	0.3835 (4)	0.2420 (3)	0.0279 (13)	
O12	0.4229 (4)	0.3040 (3)	0.1939 (2)	0.0201 (11)	
O13	0.1525 (4)	0.1935 (4)	0.2509 (2)	0.0266 (13)	
O14	0.4137 (4)	0.2615 (3)	0.4247 (2)	0.0250 (12)	
O15	0.4878 (4)	0.3109 (3)	0.3253 (2)	0.0232 (12)	
O16	0.3855 (4)	0.1555 (3)	0.1950 (2)	0.0262 (12)	
O17	0.4274 (5)	0.0358 (3)	0.1872 (3)	0.0278 (13)	
O18	0.6999 (4)	0.3596 (3)	0.3154 (3)	0.0249 (12)	
O19	0.7094 (5)	0.4754 (4)	0.2770 (3)	0.0287 (13)	
O21	1.0082 (8)	0.7575 (6)	0.1173 (4)	0.077 (3)	
N1	0.6115 (5)	0.2131 (4)	0.3736 (3)	0.0217 (14)	
N2	0.6810 (5)	0.1129 (4)	0.2135 (3)	0.0236 (14)	
N3	0.7409 (5)	0.3353 (4)	0.1536 (3)	0.0243 (15)	
N4	0.4572 (5)	0.3925 (4)	0.2015 (3)	0.0193 (13)	
N5	0.3687 (5)	0.2698 (4)	0.3167 (3)	0.0209 (14)	
N6	0.5978 (5)	0.4465 (4)	0.4367 (3)	0.0266 (15)	
N7	0.5869 (6)	0.5728 (4)	0.4346 (3)	0.0285 (16)	
N8	0.4444 (6)	0.0020 (4)	0.3156 (3)	0.0278 (16)	
N9	0.2710 (6)	−0.0377 (5)	0.3401 (4)	0.0333 (18)	
N12	0.5058 (6)	0.3922 (4)	0.0623 (3)	0.0261 (15)	
N13	0.3696 (6)	0.3739 (5)	−0.0245 (4)	0.0350 (18)	
N14	0.1925 (5)	0.2156 (4)	0.1289 (3)	0.0222 (14)	
N15	0.0488 (5)	0.1584 (4)	0.0433 (3)	0.0277 (16)	
N17	1.1778 (9)	0.7400 (6)	0.0974 (5)	0.055 (2)	
C1	0.7412 (7)	0.2946 (5)	0.5097 (4)	0.0273 (18)	
C2	0.8089 (7)	0.3292 (6)	0.5748 (4)	0.033 (2)	
H2	0.8052	0.3805	0.6021	0.040*	
C3	0.8807 (8)	0.2901 (6)	0.6000 (5)	0.039 (2)	
H3	0.9275	0.3149	0.6438	0.046*	
C4	0.8839 (7)	0.2147 (6)	0.5611 (4)	0.036 (2)	
H4	0.9330	0.1874	0.5783	0.044*	
C5	0.8169 (7)	0.1785 (6)	0.4979 (4)	0.030 (2)	
H5	0.8186	0.1257	0.4723	0.036*	
C6	0.7465 (7)	0.2181 (5)	0.4705 (4)	0.0272 (18)	
C7	0.6808 (6)	0.1773 (5)	0.4001 (4)	0.0244 (17)	
C8	0.6933 (7)	−0.0526 (5)	0.2201 (4)	0.0218 (17)	
C9	0.7137 (7)	−0.1284 (5)	0.2253 (4)	0.0277 (19)	
H9	0.6648	−0.1667	0.2454	0.033*	
C10	0.8059 (8)	−0.1473 (5)	0.2007 (4)	0.031 (2)	
H10	0.8187	−0.1988	0.2042	0.037*	
C11	0.8785 (7)	−0.0923 (5)	0.1716 (4)	0.031 (2)	
H11	0.9408	−0.1058	0.1552	0.037*	
C12	0.8599 (7)	−0.0184 (5)	0.1666 (4)	0.0256 (18)	
H12	0.9101	0.0190	0.1464	0.031*	
C13	0.7674 (7)	0.0042 (5)	0.1907 (4)	0.0256 (18)	
C14	0.7568 (6)	0.0855 (5)	0.1844 (4)	0.0215 (16)	
C15	0.9623 (7)	0.3627 (5)	0.1109 (4)	0.0240 (17)	
C16	1.0757 (7)	0.3848 (6)	0.0985 (5)	0.036 (2)	
H16	1.1086	0.3418	0.0847	0.043*	
C17	1.1388 (8)	0.4704 (6)	0.1067 (5)	0.040 (2)	
H17	1.2154	0.4856	0.0989	0.047*	
C18	1.0924 (8)	0.5327 (6)	0.1258 (6)	0.046 (3)	
H18	1.1366	0.5908	0.1314	0.055*	
C19	0.9814 (8)	0.5116 (6)	0.1371 (5)	0.037 (2)	
H19	0.9492	0.5553	0.1500	0.045*	
C20	0.9156 (7)	0.4264 (5)	0.1299 (4)	0.0264 (18)	
C21	0.7958 (7)	0.4102 (5)	0.1423 (4)	0.0265 (18)	
C22	0.5060 (7)	0.5713 (5)	0.2107 (4)	0.0286 (19)	
C23	0.5196 (8)	0.6574 (5)	0.2185 (5)	0.036 (2)	
H23	0.5829	0.6921	0.2042	0.044*	
C24	0.4455 (8)	0.6936 (6)	0.2458 (5)	0.041 (2)	
H24	0.4584	0.7527	0.2507	0.050*	
C25	0.3511 (8)	0.6445 (6)	0.2665 (5)	0.038 (2)	
H25	0.2998	0.6698	0.2858	0.046*	
C26	0.3327 (8)	0.5587 (6)	0.2587 (4)	0.033 (2)	
H26	0.2671	0.5248	0.2717	0.040*	
C27	0.4097 (7)	0.5201 (5)	0.2316 (4)	0.0271 (19)	
C28	0.3839 (7)	0.4280 (5)	0.2256 (4)	0.0266 (18)	
C29	0.1312 (7)	0.1794 (5)	0.3100 (4)	0.0247 (18)	
C30	0.0158 (7)	0.1417 (6)	0.3126 (4)	0.032 (2)	
H30	−0.0420	0.1291	0.2738	0.038*	
C31	−0.0157 (7)	0.1224 (6)	0.3711 (4)	0.032 (2)	
H31	−0.0944	0.0962	0.3721	0.039*	
C32	0.0687 (8)	0.1416 (6)	0.4288 (4)	0.039 (2)	
H32	0.0474	0.1284	0.4689	0.046*	
C33	0.1825 (7)	0.1797 (6)	0.4270 (4)	0.031 (2)	
H33	0.2400	0.1918	0.4658	0.038*	
C34	0.2143 (7)	0.2008 (5)	0.3685 (4)	0.0253 (18)	
C35	0.3384 (6)	0.2455 (5)	0.3703 (4)	0.0224 (17)	
C36	0.3540 (8)	0.0246 (6)	0.3266 (4)	0.033 (2)	
H36	0.3484	0.0787	0.3252	0.040*	
C37	0.4177 (9)	−0.0790 (6)	0.3249 (5)	0.039 (2)	
H37	0.4670	−0.1123	0.3215	0.047*	
C38	0.3104 (9)	−0.1046 (6)	0.3395 (5)	0.043 (3)	
H38	0.2713	−0.1578	0.3476	0.051*	
C39	0.1575 (8)	−0.0355 (7)	0.3518 (6)	0.046 (3)	
H39A	0.1457	0.0152	0.3412	0.070*	
H39B	0.1533	−0.0333	0.3991	0.070*	
H39C	0.0976	−0.0868	0.3227	0.070*	
C44	0.4266 (7)	0.4216 (6)	0.0375 (4)	0.032 (2)	
H44	0.4119	0.4705	0.0609	0.039*	
C45	0.4990 (8)	0.3220 (6)	0.0108 (4)	0.038 (2)	
H45	0.5459	0.2873	0.0125	0.045*	
C46	0.4142 (9)	0.3109 (7)	−0.0423 (5)	0.043 (2)	
H46	0.3912	0.2676	−0.0835	0.051*	
C47	0.2743 (9)	0.3887 (7)	−0.0654 (5)	0.049 (3)	
H47A	0.2928	0.3932	−0.1095	0.074*	
H47B	0.2644	0.4418	−0.0420	0.074*	
H47C	0.2028	0.3410	−0.0715	0.074*	
C48	0.0787 (6)	0.1769 (5)	0.1098 (4)	0.0254 (18)	
H48	0.0268	0.1646	0.1396	0.030*	
C49	0.2341 (6)	0.2238 (5)	0.0704 (4)	0.0253 (18)	
H49	0.3120	0.2503	0.0677	0.030*	
C50	0.1447 (8)	0.1875 (6)	0.0180 (4)	0.037 (2)	
H50	0.1481	0.1832	−0.0282	0.044*	
C51	−0.0700 (7)	0.1133 (6)	0.0044 (4)	0.035 (2)	
H51A	−0.1240	0.1078	0.0355	0.053*	
H51B	−0.0758	0.0565	−0.0217	0.053*	
H51C	−0.0888	0.1460	−0.0265	0.053*	
C52	0.5556 (7)	0.4904 (5)	0.4005 (4)	0.0287 (18)	
H52	0.5096	0.4666	0.3565	0.034*	
C53	0.6594 (7)	0.5046 (5)	0.4962 (4)	0.033 (2)	
H53	0.7000	0.4921	0.5324	0.040*	
C54	0.6531 (7)	0.5821 (5)	0.4953 (4)	0.034 (2)	
H54	0.6879	0.6334	0.5301	0.041*	
C55	0.5553 (11)	0.6383 (6)	0.4112 (5)	0.051 (3)	
H55A	0.6230	0.6901	0.4213	0.077*	
H55B	0.4952	0.6510	0.4339	0.077*	
H55C	0.5264	0.6185	0.3623	0.077*	
C56	0.3561 (6)	0.0741 (5)	0.1823 (4)	0.0237 (17)	
C57	0.2298 (7)	0.0248 (5)	0.1627 (5)	0.035 (2)	
H57A	0.1966	0.0393	0.1233	0.052*	
H57B	0.1920	0.0394	0.2004	0.052*	
H57C	0.2180	−0.0365	0.1516	0.052*	
C58	0.7437 (7)	0.4380 (5)	0.3170 (4)	0.0276 (19)	
C59	0.8493 (8)	0.4901 (6)	0.3721 (5)	0.040 (2)	
H59A	0.9188	0.4980	0.3526	0.060*	
H59B	0.8535	0.4597	0.4072	0.060*	
H59C	0.8434	0.5459	0.3919	0.060*	
C63	1.0639 (11)	0.7248 (8)	0.0824 (6)	0.058 (3)	
H63	1.0231	0.6850	0.0408	0.070*	
C64	1.2447 (11)	0.7980 (8)	0.1637 (6)	0.064 (3)	
H64A	1.2936	0.7710	0.1848	0.096*	
H64B	1.1916	0.8094	0.1930	0.096*	
H64C	1.2929	0.8518	0.1569	0.096*	
C65	1.2376 (12)	0.7004 (9)	0.0527 (7)	0.077 (4)	
H65A	1.1816	0.6534	0.0171	0.115*	
H65B	1.2881	0.6783	0.0781	0.115*	
H65C	1.2838	0.7426	0.0322	0.115*	
O20	0.6594 (5)	0.1644 (4)	0.0531 (3)	0.0323 (14)	
C60	0.5545 (7)	0.1347 (5)	0.0516 (4)	0.032 (2)	
H60	0.5291	0.1407	0.0934	0.038*	
N16	0.4749 (6)	0.0948 (4)	−0.0048 (3)	0.0290 (16)	
C61	0.5092 (8)	0.0841 (7)	−0.0693 (5)	0.050 (3)	
H61A	0.4738	0.0243	−0.0953	0.076*	
H61B	0.5932	0.1000	−0.0626	0.076*	
H61C	0.4840	0.1207	−0.0941	0.076*	
C62	0.3540 (7)	0.0622 (7)	−0.0048 (5)	0.046 (3)	
H62A	0.3124	0.0864	−0.0340	0.069*	
H62B	0.3409	0.0782	0.0413	0.069*	
H62C	0.3262	−0.0001	−0.0215	0.069*	
N10	0.926 (2)	0.2986 (16)	0.2537 (10)	0.018 (4)	0.61983 (2000)
C40	0.9050 (18)	0.298 (2)	0.3148 (9)	0.026 (4)	0.61983 (2000)
H40	0.8322	0.2711	0.3245	0.031*	0.61983 (2000)
C41	1.0418 (11)	0.3449 (9)	0.2625 (7)	0.032 (3)	0.61983 (2000)
H41	1.0806	0.3584	0.2272	0.038*	0.61983 (2000)
C42	1.0926 (18)	0.3686 (12)	0.3292 (11)	0.031 (4)	0.61983 (2000)
H42	1.1719	0.3964	0.3488	0.037*	0.61983 (2000)
N11	1.0021 (17)	0.3431 (17)	0.3619 (9)	0.032 (3)	0.61983 (2000)
C43	1.012 (3)	0.352 (3)	0.4357 (11)	0.036 (4)	0.61983 (2000)
H43A	0.9349	0.3336	0.4463	0.055*	0.61983 (2000)
H43B	1.0529	0.4115	0.4602	0.055*	0.61983 (2000)
H43C	1.0547	0.3160	0.4491	0.055*	0.61983 (2000)
O22	0.919 (3)	0.304 (2)	0.2376 (13)	0.024 (7)	0.38017 (2000)
C40B	0.918 (3)	0.303 (3)	0.2977 (15)	0.026 (4)	0.38017 (2000)
H40B	0.8482	0.2713	0.3083	0.031*	0.38017 (2000)
N11B	1.009 (3)	0.343 (3)	0.3485 (12)	0.032 (3)	0.38017 (2000)
C42B	1.113 (3)	0.398 (2)	0.3362 (19)	0.031 (4)	0.38017 (2000)
H42A	1.1787	0.3837	0.3544	0.037*	0.38017 (2000)
H42B	1.1227	0.4574	0.3583	0.037*	0.38017 (2000)
H42C	1.1076	0.3912	0.2875	0.037*	0.38017 (2000)
C43B	1.007 (6)	0.343 (5)	0.4180 (15)	0.036 (4)	0.38017 (2000)
H43D	0.9357	0.3002	0.4208	0.055*	0.38017 (2000)
H43E	1.0116	0.3994	0.4436	0.055*	0.38017 (2000)
H43F	1.0732	0.3287	0.4367	0.055*	0.38017 (2000)
C67	0.524 (2)	0.885 (2)	0.4934 (15)	0.056 (9)	0.50926 (1100)
H67A	0.5447	0.8590	0.5288	0.084*	0.50926 (1100)
H67B	0.4699	0.8405	0.4553	0.084*	0.50926 (1100)
H67C	0.4871	0.9263	0.5114	0.084*	0.50926 (1100)
C68	0.632 (4)	0.931 (2)	0.470 (4)	0.070 (6)	0.50926 (1100)
H68A	0.6130	0.9652	0.4399	0.083*	0.50926 (1100)
H68B	0.6907	0.9695	0.5091	0.083*	0.50926 (1100)
O23	0.6761 (11)	0.8707 (9)	0.4341 (7)	0.042 (4)	0.50926 (1100)
C69	0.779 (2)	0.9130 (19)	0.4118 (13)	0.049 (5)	0.50926 (1100)
H69A	0.8372	0.9545	0.4504	0.059*	0.50926 (1100)
H69B	0.7610	0.9435	0.3784	0.059*	0.50926 (1100)
C70	0.823 (2)	0.8415 (19)	0.3795 (12)	0.072 (8)	0.50926 (1100)
H70A	0.7729	0.8086	0.3359	0.108*	0.50926 (1100)
H70B	0.8232	0.8037	0.4093	0.108*	0.50926 (1100)
H70C	0.9017	0.8668	0.3727	0.108*	0.50926 (1100)
C67B	0.527 (2)	0.862 (2)	0.4667 (16)	0.059 (9)	0.49074 (1100)
H67D	0.5568	0.8294	0.4938	0.089*	0.49074 (1100)
H67E	0.5090	0.8312	0.4189	0.089*	0.49074 (1100)
H67F	0.4572	0.8692	0.4808	0.089*	0.49074 (1100)
C68B	0.618 (4)	0.949 (2)	0.477 (4)	0.070 (6)	0.49074 (1100)
H68C	0.5947	0.9773	0.4431	0.083*	0.49074 (1100)
H68D	0.6219	0.9855	0.5224	0.083*	0.49074 (1100)
O23B	0.7272 (15)	0.9433 (12)	0.4718 (7)	0.063 (5)	0.49074 (1100)
C69B	0.741 (2)	0.920 (2)	0.4032 (12)	0.049 (5)	0.49074 (1100)
H69C	0.7267	0.9614	0.3789	0.059*	0.49074 (1100)
H69D	0.6857	0.8625	0.3796	0.059*	0.49074 (1100)
C70B	0.8652 (18)	0.9207 (19)	0.4061 (11)	0.062 (8)	0.49074 (1100)
H70D	0.8836	0.8909	0.4394	0.092*	0.49074 (1100)
H70E	0.9178	0.9798	0.4192	0.092*	0.49074 (1100)
H70F	0.8735	0.8917	0.3616	0.092*	0.49074 (1100)
O24	1.033 (2)	0.7637 (18)	0.2477 (12)	0.069 (5)	0.49074 (1100)
H24A	0.98 (2)	0.716 (6)	0.246 (14)	0.104*	0.49074 (1100)
H24B	1.066 (5)	0.769 (19)	0.215 (3)	0.104*	0.49074 (1100)
O25	0.8511 (16)	0.6404 (11)	0.2802 (10)	0.057 (5)	0.49074 (1100)
H25A	0.8459	0.5927	0.2873	0.085*	0.49074 (1100)
C72	0.8032 (19)	0.6824 (14)	0.3257 (10)	0.050 (6)	0.49074 (1100)
H72A	0.8101	0.7384	0.3179	0.075*	0.49074 (1100)
H72B	0.8432	0.6899	0.3715	0.075*	0.49074 (1100)
H72C	0.7217	0.6492	0.3205	0.075*	0.49074 (1100)
C71	0.880 (2)	0.6747 (14)	0.2613 (11)	0.046 (6)	0.50926 (1100)
H71A	0.8977	0.6216	0.2546	0.069*	0.50926 (1100)
H71B	0.8191	0.6695	0.2241	0.069*	0.50926 (1100)
H71C	0.8529	0.6855	0.3041	0.069*	0.50926 (1100)
O26	0.980 (2)	0.7439 (17)	0.2632 (12)	0.069 (5)	0.50926 (1100)
H26A	0.9827	0.7503	0.2241	0.104*	0.50926 (1100)

### Atomic displacement parameters (Å^2^)

**Table:**

	*U*^11^	*U*^22^	*U*^33^	*U*^12^	*U*^13^	*U*^23^
Mn1	0.0181 (6)	0.0201 (6)	0.0178 (6)	0.0066 (5)	0.0045 (4)	0.0000 (4)
Mn2	0.0220 (6)	0.0225 (6)	0.0200 (6)	0.0084 (5)	0.0012 (5)	−0.0033 (5)
Mn3	0.0233 (6)	0.0202 (6)	0.0210 (6)	0.0079 (5)	0.0066 (5)	−0.0002 (5)
Mn4	0.0236 (6)	0.0234 (6)	0.0313 (7)	0.0111 (5)	0.0132 (5)	0.0063 (5)
Mn5	0.0222 (6)	0.0208 (6)	0.0266 (6)	0.0102 (5)	0.0097 (5)	0.0039 (5)
Mn6	0.0177 (6)	0.0257 (6)	0.0178 (6)	0.0059 (5)	0.0040 (4)	0.0004 (5)
O1	0.039 (3)	0.030 (3)	0.022 (3)	0.019 (3)	−0.006 (2)	−0.006 (2)
O2	0.030 (3)	0.025 (3)	0.025 (3)	0.014 (2)	0.003 (2)	0.001 (2)
O3	0.025 (3)	0.022 (3)	0.017 (3)	0.006 (2)	0.003 (2)	−0.007 (2)
O4	0.024 (3)	0.020 (3)	0.037 (3)	0.009 (2)	0.010 (3)	0.003 (2)
O5	0.025 (3)	0.017 (3)	0.028 (3)	0.006 (2)	0.010 (2)	0.006 (2)
O6	0.028 (3)	0.017 (3)	0.022 (3)	0.005 (2)	0.010 (2)	0.004 (2)
O7	0.030 (3)	0.028 (3)	0.040 (3)	0.010 (3)	0.021 (3)	0.005 (3)
O8	0.022 (3)	0.027 (3)	0.035 (3)	0.010 (2)	0.012 (2)	0.004 (3)
O9	0.020 (3)	0.022 (3)	0.024 (3)	0.008 (2)	0.006 (2)	0.002 (2)
O10	0.027 (3)	0.030 (3)	0.030 (3)	0.016 (3)	0.012 (3)	0.006 (3)
O11	0.025 (3)	0.029 (3)	0.032 (3)	0.014 (2)	0.010 (2)	0.003 (2)
O12	0.018 (3)	0.020 (3)	0.020 (3)	0.005 (2)	0.005 (2)	0.003 (2)
O13	0.027 (3)	0.033 (3)	0.014 (3)	0.003 (2)	0.004 (2)	0.004 (2)
O14	0.023 (3)	0.028 (3)	0.020 (3)	0.012 (2)	0.000 (2)	−0.002 (2)
O15	0.016 (2)	0.024 (3)	0.024 (3)	0.004 (2)	0.006 (2)	−0.002 (2)
O16	0.020 (3)	0.028 (3)	0.022 (3)	0.007 (2)	−0.003 (2)	−0.005 (2)
O17	0.031 (3)	0.025 (3)	0.026 (3)	0.012 (2)	0.006 (2)	−0.001 (2)
O18	0.019 (3)	0.023 (3)	0.023 (3)	0.002 (2)	0.001 (2)	−0.005 (2)
O19	0.032 (3)	0.029 (3)	0.026 (3)	0.012 (3)	0.006 (2)	0.005 (2)
O21	0.068 (6)	0.097 (7)	0.063 (5)	0.054 (5)	−0.009 (5)	−0.011 (5)
N1	0.020 (3)	0.024 (3)	0.017 (3)	0.005 (3)	0.007 (3)	−0.003 (3)
N2	0.022 (3)	0.014 (3)	0.032 (4)	0.005 (3)	0.006 (3)	0.000 (3)
N3	0.021 (3)	0.024 (4)	0.031 (4)	0.011 (3)	0.012 (3)	0.006 (3)
N4	0.018 (3)	0.017 (3)	0.024 (3)	0.008 (3)	0.009 (3)	0.004 (3)
N5	0.013 (3)	0.019 (3)	0.025 (3)	0.000 (2)	0.007 (2)	0.000 (3)
N6	0.026 (3)	0.024 (4)	0.025 (3)	0.008 (3)	0.005 (3)	−0.003 (3)
N7	0.041 (4)	0.021 (3)	0.024 (3)	0.013 (3)	0.006 (3)	0.002 (3)
N8	0.033 (4)	0.023 (4)	0.024 (4)	0.005 (3)	0.012 (3)	0.001 (3)
N9	0.034 (4)	0.035 (4)	0.032 (4)	0.010 (3)	0.016 (3)	0.008 (3)
N12	0.026 (4)	0.032 (4)	0.023 (3)	0.013 (3)	0.009 (3)	0.005 (3)
N13	0.030 (4)	0.049 (5)	0.035 (4)	0.022 (4)	0.011 (3)	0.017 (4)
N14	0.019 (3)	0.027 (4)	0.021 (3)	0.010 (3)	0.008 (3)	0.003 (3)
N15	0.023 (3)	0.031 (4)	0.022 (3)	0.010 (3)	−0.002 (3)	−0.007 (3)
N17	0.063 (6)	0.058 (6)	0.051 (6)	0.025 (5)	0.017 (5)	0.021 (5)
C1	0.026 (4)	0.022 (4)	0.029 (4)	0.005 (3)	0.005 (3)	0.002 (3)
C2	0.029 (5)	0.039 (5)	0.026 (4)	0.011 (4)	−0.002 (4)	−0.001 (4)
C3	0.027 (5)	0.054 (6)	0.031 (5)	0.013 (4)	−0.003 (4)	0.007 (4)
C4	0.024 (4)	0.051 (6)	0.033 (5)	0.013 (4)	0.008 (4)	0.007 (4)
C5	0.026 (4)	0.036 (5)	0.034 (5)	0.015 (4)	0.014 (4)	0.008 (4)
C6	0.018 (4)	0.029 (5)	0.028 (4)	0.001 (3)	0.006 (3)	0.003 (4)
C7	0.017 (4)	0.023 (4)	0.031 (4)	0.003 (3)	0.012 (3)	0.004 (3)
C8	0.030 (4)	0.016 (4)	0.019 (4)	0.011 (3)	0.002 (3)	0.000 (3)
C9	0.033 (5)	0.021 (4)	0.026 (4)	0.008 (4)	0.007 (4)	0.002 (3)
C10	0.038 (5)	0.019 (4)	0.037 (5)	0.011 (4)	0.010 (4)	0.007 (4)
C11	0.032 (5)	0.026 (5)	0.032 (5)	0.015 (4)	0.007 (4)	−0.003 (4)
C12	0.022 (4)	0.023 (4)	0.029 (4)	0.008 (3)	0.007 (3)	−0.001 (3)
C13	0.026 (4)	0.024 (4)	0.024 (4)	0.011 (3)	0.007 (3)	−0.004 (3)
C14	0.022 (4)	0.017 (4)	0.022 (4)	0.002 (3)	0.002 (3)	0.004 (3)
C15	0.020 (4)	0.031 (4)	0.024 (4)	0.008 (3)	0.011 (3)	0.010 (3)
C16	0.028 (5)	0.046 (6)	0.044 (6)	0.023 (4)	0.014 (4)	0.012 (4)
C17	0.025 (5)	0.043 (6)	0.060 (7)	0.015 (4)	0.020 (4)	0.020 (5)
C18	0.031 (5)	0.033 (5)	0.072 (8)	0.003 (4)	0.019 (5)	0.016 (5)
C19	0.032 (5)	0.024 (5)	0.062 (7)	0.013 (4)	0.014 (5)	0.016 (4)
C20	0.020 (4)	0.030 (5)	0.031 (5)	0.011 (3)	0.008 (3)	0.008 (4)
C21	0.025 (4)	0.030 (5)	0.023 (4)	0.012 (4)	0.006 (3)	−0.002 (3)
C22	0.030 (4)	0.030 (5)	0.030 (4)	0.019 (4)	0.006 (4)	0.003 (4)
C23	0.045 (6)	0.017 (4)	0.046 (6)	0.009 (4)	0.017 (5)	0.005 (4)
C24	0.041 (6)	0.032 (5)	0.054 (6)	0.019 (4)	0.015 (5)	0.005 (5)
C25	0.037 (5)	0.036 (5)	0.051 (6)	0.024 (4)	0.021 (5)	0.006 (4)
C26	0.032 (5)	0.031 (5)	0.039 (5)	0.017 (4)	0.011 (4)	0.001 (4)
C27	0.022 (4)	0.028 (4)	0.029 (4)	0.014 (3)	0.004 (3)	−0.004 (3)
C28	0.020 (4)	0.032 (5)	0.026 (4)	0.010 (3)	0.001 (3)	0.002 (4)
C29	0.025 (4)	0.023 (4)	0.022 (4)	0.006 (3)	0.004 (3)	0.001 (3)
C30	0.027 (4)	0.036 (5)	0.021 (4)	0.003 (4)	0.006 (3)	−0.003 (4)
C31	0.021 (4)	0.041 (5)	0.030 (5)	0.005 (4)	0.009 (4)	0.006 (4)
C32	0.029 (5)	0.056 (6)	0.026 (5)	0.009 (4)	0.006 (4)	0.008 (4)
C33	0.032 (5)	0.036 (5)	0.026 (4)	0.011 (4)	0.009 (4)	0.006 (4)
C34	0.023 (4)	0.028 (4)	0.025 (4)	0.012 (3)	0.008 (3)	−0.002 (3)
C35	0.023 (4)	0.021 (4)	0.019 (4)	0.010 (3)	0.007 (3)	−0.006 (3)
C36	0.033 (5)	0.031 (5)	0.038 (5)	0.012 (4)	0.011 (4)	0.009 (4)
C37	0.048 (6)	0.031 (5)	0.048 (6)	0.019 (4)	0.030 (5)	0.010 (4)
C38	0.058 (7)	0.028 (5)	0.057 (6)	0.022 (5)	0.036 (5)	0.014 (5)
C39	0.036 (5)	0.044 (6)	0.068 (7)	0.017 (5)	0.026 (5)	0.020 (5)
C44	0.029 (5)	0.042 (5)	0.031 (5)	0.019 (4)	0.009 (4)	0.009 (4)
C45	0.047 (6)	0.043 (6)	0.027 (5)	0.021 (5)	0.014 (4)	0.006 (4)
C46	0.050 (6)	0.049 (6)	0.030 (5)	0.021 (5)	0.010 (5)	0.003 (4)
C47	0.036 (6)	0.067 (7)	0.053 (7)	0.021 (5)	0.005 (5)	0.031 (6)
C48	0.019 (4)	0.034 (5)	0.023 (4)	0.015 (3)	0.003 (3)	0.000 (3)
C49	0.017 (4)	0.030 (4)	0.028 (4)	0.010 (3)	0.004 (3)	0.001 (3)
C50	0.044 (5)	0.050 (6)	0.017 (4)	0.018 (5)	0.009 (4)	0.003 (4)
C51	0.037 (5)	0.033 (5)	0.022 (4)	0.001 (4)	0.001 (4)	−0.004 (4)
C52	0.036 (5)	0.024 (4)	0.022 (4)	0.010 (4)	0.003 (3)	0.000 (3)
C53	0.029 (4)	0.035 (5)	0.025 (4)	0.010 (4)	−0.004 (3)	−0.008 (4)
C54	0.035 (5)	0.029 (5)	0.028 (4)	0.007 (4)	0.008 (4)	−0.009 (4)
C55	0.091 (9)	0.028 (5)	0.037 (5)	0.029 (5)	0.002 (5)	0.004 (4)
C56	0.024 (4)	0.025 (4)	0.015 (4)	0.004 (3)	0.003 (3)	−0.003 (3)
C57	0.025 (4)	0.021 (4)	0.044 (5)	0.001 (3)	−0.003 (4)	−0.006 (4)
C58	0.027 (4)	0.028 (5)	0.019 (4)	0.004 (4)	0.010 (3)	−0.007 (3)
C59	0.035 (5)	0.032 (5)	0.043 (5)	0.011 (4)	−0.008 (4)	−0.001 (4)
C63	0.060 (8)	0.056 (7)	0.053 (7)	0.018 (6)	−0.006 (6)	0.012 (6)
C64	0.063 (8)	0.056 (8)	0.069 (8)	0.014 (6)	0.003 (7)	0.018 (6)
C65	0.082 (10)	0.095 (11)	0.075 (9)	0.036 (8)	0.050 (8)	0.039 (8)
O20	0.029 (3)	0.036 (3)	0.030 (3)	0.010 (3)	0.009 (3)	0.004 (3)
C60	0.041 (5)	0.028 (4)	0.020 (4)	0.017 (4)	−0.006 (4)	−0.007 (3)
N16	0.028 (4)	0.033 (4)	0.018 (3)	0.009 (3)	0.000 (3)	−0.005 (3)
C61	0.038 (5)	0.055 (7)	0.050 (6)	0.013 (5)	0.015 (5)	−0.003 (5)
C62	0.030 (5)	0.059 (7)	0.041 (5)	0.015 (5)	0.013 (4)	−0.006 (5)
N10	0.028 (7)	0.025 (7)	0.010 (8)	0.017 (5)	0.002 (6)	0.010 (6)
C40	0.019 (6)	0.030 (5)	0.027 (8)	0.009 (5)	0.001 (6)	0.007 (8)
C41	0.026 (4)	0.032 (4)	0.030 (6)	0.011 (3)	0.004 (4)	−0.005 (5)
C42	0.028 (7)	0.031 (10)	0.030 (6)	0.009 (7)	0.006 (5)	0.000 (7)
N11	0.026 (4)	0.032 (4)	0.030 (6)	0.011 (3)	0.004 (4)	−0.005 (5)
C43	0.036 (6)	0.048 (12)	0.015 (11)	0.010 (6)	0.005 (11)	−0.007 (13)
O22	0.018 (9)	0.028 (10)	0.024 (11)	0.004 (7)	−0.005 (8)	0.009 (8)
C40B	0.019 (6)	0.030 (5)	0.027 (8)	0.009 (5)	0.001 (6)	0.007 (8)
N11B	0.026 (4)	0.032 (4)	0.030 (6)	0.011 (3)	0.004 (4)	−0.005 (5)
C42B	0.028 (7)	0.031 (10)	0.030 (6)	0.009 (7)	0.006 (5)	0.000 (7)
C43B	0.036 (6)	0.048 (12)	0.015 (11)	0.010 (6)	0.005 (11)	−0.007 (13)
C67	0.064 (15)	0.063 (16)	0.051 (15)	0.019 (12)	0.036 (12)	0.025 (12)
C68	0.074 (14)	0.084 (17)	0.051 (16)	0.024 (12)	0.018 (9)	0.019 (17)
O23	0.044 (8)	0.041 (8)	0.044 (8)	0.017 (7)	0.018 (7)	0.008 (7)
C69	0.056 (11)	0.059 (7)	0.036 (7)	0.019 (7)	0.009 (8)	0.020 (5)
C70	0.054 (15)	0.12 (3)	0.044 (14)	0.032 (16)	0.016 (12)	0.016 (16)
C67B	0.072 (19)	0.07 (2)	0.07 (2)	0.049 (17)	0.037 (18)	0.037 (18)
C68B	0.074 (14)	0.084 (17)	0.051 (16)	0.024 (12)	0.018 (9)	0.019 (17)
O23B	0.070 (12)	0.085 (14)	0.038 (9)	0.031 (11)	0.010 (9)	0.015 (9)
C69B	0.056 (11)	0.059 (7)	0.036 (7)	0.019 (7)	0.009 (8)	0.020 (5)
C70B	0.040 (13)	0.09 (2)	0.038 (13)	0.011 (13)	−0.001 (11)	0.012 (14)
O24	0.064 (14)	0.079 (12)	0.057 (11)	0.020 (12)	0.002 (9)	0.015 (8)
O25	0.062 (11)	0.037 (9)	0.069 (12)	0.010 (8)	0.013 (9)	0.022 (8)
C72	0.052 (13)	0.053 (14)	0.037 (12)	0.004 (11)	0.003 (10)	0.016 (10)
C71	0.071 (17)	0.030 (12)	0.031 (12)	0.017 (12)	−0.008 (11)	0.007 (9)
O26	0.064 (14)	0.079 (12)	0.057 (11)	0.020 (12)	0.002 (9)	0.015 (8)

### Geometric parameters (Å, º)

**Table:**

Mn1—O18	2.211 (5)	C29—C30	1.396 (11)
Mn1—O6	2.226 (5)	C29—C34	1.399 (11)
Mn1—O15	2.237 (5)	C30—C31	1.387 (12)
Mn1—O9	2.244 (5)	C30—H30	0.9500
Mn1—O12	2.246 (5)	C31—C32	1.400 (12)
Mn1—O3	2.265 (6)	C31—H31	0.9500
Mn1—O16	2.280 (5)	C32—C33	1.375 (12)
Mn2—O1	1.851 (5)	C32—H32	0.9500
Mn2—O15	1.889 (5)	C33—C34	1.397 (12)
Mn2—N1	1.970 (6)	C33—H33	0.9500
Mn2—N6	2.045 (6)	C34—C35	1.492 (10)
Mn2—O14	2.132 (5)	C36—H36	0.9500
Mn3—O4	1.874 (5)	C37—C38	1.362 (13)
Mn3—O3	1.901 (5)	C37—H37	0.9500
Mn3—N2	2.005 (7)	C38—H38	0.9500
Mn3—N8	2.067 (7)	C39—H39A	0.9800
Mn3—O2	2.119 (5)	C39—H39B	0.9800
Mn3—O17	2.245 (6)	C39—H39C	0.9800
Mn4—O7	1.869 (6)	C44—H44	0.9500
Mn4—O6	1.912 (5)	C45—C46	1.368 (13)
Mn4—O5	1.951 (5)	C45—H45	0.9500
Mn4—N3	1.957 (7)	C46—H46	0.9500
Mn4—O22	2.08 (3)	C47—H47A	0.9800
Mn4—O20	2.308 (6)	C47—H47B	0.9800
Mn4—N10	2.35 (2)	C47—H47C	0.9800
Mn5—O10	1.871 (6)	C48—H48	0.9500
Mn5—O9	1.938 (5)	C49—C50	1.345 (11)
Mn5—O8	1.942 (5)	C49—H49	0.9500
Mn5—N4	1.945 (6)	C50—H50	0.9500
Mn5—O19	2.267 (6)	C51—H51A	0.9800
Mn5—N12	2.282 (7)	C51—H51B	0.9800
Mn6—O13	1.864 (5)	C51—H51C	0.9800
Mn6—O12	1.916 (5)	C52—H52	0.9500
Mn6—N5	1.967 (6)	C53—C54	1.349 (12)
Mn6—N14	2.043 (6)	C53—H53	0.9500
Mn6—O11	2.144 (6)	C54—H54	0.9500
Mn6—O16	2.379 (5)	C55—H55A	0.9800
O1—C1	1.337 (10)	C55—H55B	0.9800
O2—C7	1.279 (9)	C55—H55C	0.9800
O3—N1	1.419 (7)	C56—C57	1.498 (10)
O4—C8	1.321 (9)	C57—H57A	0.9800
O5—C14	1.310 (9)	C57—H57B	0.9800
O6—N2	1.434 (8)	C57—H57C	0.9800
O7—C15	1.343 (10)	C58—C59	1.526 (11)
O8—C21	1.288 (10)	C59—H59A	0.9800
O9—N3	1.406 (8)	C59—H59B	0.9800
O10—C22	1.332 (9)	C59—H59C	0.9800
O11—C28	1.284 (10)	C63—H63	0.9500
O12—N4	1.404 (7)	C64—H64A	0.9800
O13—C29	1.333 (9)	C64—H64B	0.9800
O14—C35	1.296 (9)	C64—H64C	0.9800
O15—N5	1.404 (7)	C65—H65A	0.9800
O16—C56	1.281 (9)	C65—H65B	0.9800
O17—C56	1.255 (9)	C65—H65C	0.9800
O18—C58	1.272 (10)	O20—C60	1.241 (10)
O19—C58	1.254 (10)	C60—N16	1.338 (9)
O21—C63	1.219 (14)	C60—H60	0.9500
N1—C7	1.325 (10)	N16—C62	1.436 (10)
N2—C14	1.320 (9)	N16—C61	1.438 (11)
N3—C21	1.335 (10)	C61—H61A	0.9800
N4—C28	1.325 (9)	C61—H61B	0.9800
N5—C35	1.319 (10)	C61—H61C	0.9800
N6—C52	1.329 (11)	C62—H62A	0.9800
N6—C53	1.373 (9)	C62—H62B	0.9800
N7—C52	1.343 (9)	C62—H62C	0.9800
N7—C54	1.367 (11)	N10—C40	1.32 (2)
N7—C55	1.441 (11)	N10—C41	1.38 (3)
N8—C36	1.320 (11)	C40—N11	1.36 (2)
N8—C37	1.377 (11)	C40—H40	0.9500
N9—C36	1.341 (11)	C41—C42	1.37 (3)
N9—C38	1.366 (12)	C41—H41	0.9500
N9—C39	1.467 (11)	C42—N11	1.38 (2)
N12—C44	1.328 (11)	C42—H42	0.9500
N12—C45	1.395 (10)	N11—C43	1.49 (2)
N13—C44	1.344 (11)	C43—H43A	0.9800
N13—C46	1.351 (12)	C43—H43B	0.9800
N13—C47	1.489 (12)	C43—H43C	0.9800
N14—C48	1.337 (9)	O22—C40B	1.25 (2)
N14—C49	1.386 (10)	C40B—N11B	1.34 (2)
N15—C48	1.323 (10)	C40B—H40B	0.9500
N15—C50	1.355 (11)	N11B—C42B	1.43 (2)
N15—C51	1.481 (10)	N11B—C43B	1.44 (2)
N17—C63	1.344 (15)	C42B—H42A	0.9800
N17—C65	1.434 (14)	C42B—H42B	0.9800
N17—C64	1.487 (14)	C42B—H42C	0.9800
C1—C2	1.403 (11)	C43B—H43D	0.9800
C1—C6	1.405 (11)	C43B—H43E	0.9800
C2—C3	1.380 (13)	C43B—H43F	0.9800
C2—H2	0.9500	C67—C68	1.53 (2)
C3—C4	1.377 (13)	C67—H67A	0.9800
C3—H3	0.9500	C67—H67B	0.9800
C4—C5	1.370 (12)	C67—H67C	0.9800
C4—H4	0.9500	C68—O23	1.41 (3)
C5—C6	1.391 (12)	C68—H68A	0.9900
C5—H5	0.9500	C68—H68B	0.9900
C6—C7	1.489 (11)	O23—C69	1.44 (2)
C8—C9	1.409 (11)	C69—C70	1.54 (3)
C8—C13	1.412 (11)	C69—H69A	0.9900
C9—C10	1.400 (12)	C69—H69B	0.9900
C9—H9	0.9500	C70—H70A	0.9800
C10—C11	1.381 (12)	C70—H70B	0.9800
C10—H10	0.9500	C70—H70C	0.9800
C11—C12	1.367 (12)	C67B—C68B	1.53 (3)
C11—H11	0.9500	C67B—H67D	0.9800
C12—C13	1.426 (10)	C67B—H67E	0.9800
C12—H12	0.9500	C67B—H67F	0.9800
C13—C14	1.458 (11)	C68B—O23B	1.41 (3)
C15—C20	1.385 (11)	C68B—H68C	0.9900
C15—C16	1.413 (11)	C68B—H68D	0.9900
C16—C17	1.394 (13)	O23B—C69B	1.43 (2)
C16—H16	0.9500	C69B—C70B	1.54 (3)
C17—C18	1.362 (13)	C69B—H69C	0.9900
C17—H17	0.9500	C69B—H69D	0.9900
C18—C19	1.377 (12)	C70B—H70D	0.9800
C18—H18	0.9500	C70B—H70E	0.9800
C19—C20	1.400 (12)	C70B—H70F	0.9800
C19—H19	0.9500	O24—H24A	0.85 (2)
C20—C21	1.491 (11)	O24—H24B	0.85 (2)
C22—C23	1.394 (11)	O25—C72	1.36 (3)
C22—C27	1.425 (12)	O25—H25A	0.8400
C23—C24	1.365 (12)	C72—H72A	0.9800
C23—H23	0.9500	C72—H72B	0.9800
C24—C25	1.391 (13)	C72—H72C	0.9800
C24—H24	0.9500	C71—O26	1.43 (4)
C25—C26	1.378 (12)	C71—H71A	0.9800
C25—H25	0.9500	C71—H71B	0.9800
C26—C27	1.416 (11)	C71—H71C	0.9800
C26—H26	0.9500	O26—H26A	0.8400
C27—C28	1.477 (12)		
			
O18—Mn1—O6	93.13 (19)	C27—C26—H26	119.4
O18—Mn1—O15	70.27 (18)	C26—C27—C22	118.7 (8)
O6—Mn1—O15	148.3 (2)	C26—C27—C28	117.6 (8)
O18—Mn1—O9	87.6 (2)	C22—C27—C28	123.7 (7)
O6—Mn1—O9	77.54 (19)	O11—C28—N4	121.0 (7)
O15—Mn1—O9	126.44 (19)	O11—C28—C27	121.5 (7)
O18—Mn1—O12	114.48 (19)	N4—C28—C27	117.5 (7)
O6—Mn1—O12	137.17 (18)	O13—C29—C30	116.1 (7)
O15—Mn1—O12	74.31 (18)	O13—C29—C34	125.3 (7)
O9—Mn1—O12	71.99 (18)	C30—C29—C34	118.6 (8)
O18—Mn1—O3	89.1 (2)	C31—C30—C29	120.8 (8)
O6—Mn1—O3	77.94 (18)	C31—C30—H30	119.6
O15—Mn1—O3	75.07 (19)	C29—C30—H30	119.6
O9—Mn1—O3	155.02 (18)	C30—C31—C32	120.0 (8)
O12—Mn1—O3	131.25 (19)	C30—C31—H31	120.0
O18—Mn1—O16	157.79 (19)	C32—C31—H31	120.0
O6—Mn1—O16	101.13 (18)	C33—C32—C31	119.6 (9)
O15—Mn1—O16	89.02 (18)	C33—C32—H32	120.2
O9—Mn1—O16	111.98 (19)	C31—C32—H32	120.2
O12—Mn1—O16	65.01 (19)	C32—C33—C34	120.5 (8)
O3—Mn1—O16	77.50 (19)	C32—C33—H33	119.7
O1—Mn2—O15	175.9 (2)	C34—C33—H33	119.7
O1—Mn2—N1	89.4 (2)	C33—C34—C29	120.3 (7)
O15—Mn2—N1	94.2 (2)	C33—C34—C35	118.2 (7)
O1—Mn2—N6	87.0 (3)	C29—C34—C35	121.4 (7)
O15—Mn2—N6	89.0 (2)	O14—C35—N5	121.4 (7)
N1—Mn2—N6	163.1 (3)	O14—C35—C34	119.8 (7)
O1—Mn2—O14	103.4 (2)	N5—C35—C34	118.8 (7)
O15—Mn2—O14	78.2 (2)	N8—C36—N9	112.2 (8)
N1—Mn2—O14	95.8 (2)	N8—C36—H36	123.9
N6—Mn2—O14	101.1 (2)	N9—C36—H36	123.9
O4—Mn3—O3	176.2 (2)	C38—C37—N8	110.3 (8)
O4—Mn3—N2	88.5 (2)	C38—C37—H37	124.8
O3—Mn3—N2	91.5 (2)	N8—C37—H37	124.8
O4—Mn3—N8	89.7 (3)	C37—C38—N9	105.6 (8)
O3—Mn3—N8	91.0 (2)	C37—C38—H38	127.2
N2—Mn3—N8	169.5 (3)	N9—C38—H38	127.2
O4—Mn3—O2	97.5 (2)	N9—C39—H39A	109.5
O3—Mn3—O2	78.8 (2)	N9—C39—H39B	109.5
N2—Mn3—O2	99.1 (2)	H39A—C39—H39B	109.5
N8—Mn3—O2	91.4 (2)	N9—C39—H39C	109.5
O4—Mn3—O17	96.3 (2)	H39A—C39—H39C	109.5
O3—Mn3—O17	87.5 (2)	H39B—C39—H39C	109.5
N2—Mn3—O17	88.0 (2)	N12—C44—N13	112.1 (8)
N8—Mn3—O17	81.9 (2)	N12—C44—H44	124.0
O2—Mn3—O17	164.7 (2)	N13—C44—H44	124.0
O7—Mn4—O6	179.0 (2)	C46—C45—N12	109.5 (8)
O7—Mn4—O5	97.2 (2)	C46—C45—H45	125.2
O6—Mn4—O5	82.2 (2)	N12—C45—H45	125.2
O7—Mn4—N3	90.2 (3)	N13—C46—C45	106.4 (8)
O6—Mn4—N3	90.4 (2)	N13—C46—H46	126.8
O5—Mn4—N3	171.3 (2)	C45—C46—H46	126.8
O7—Mn4—O22	86.4 (10)	N13—C47—H47A	109.5
O6—Mn4—O22	94.4 (10)	N13—C47—H47B	109.5
O5—Mn4—O22	93.6 (11)	H47A—C47—H47B	109.5
N3—Mn4—O22	91.5 (11)	N13—C47—H47C	109.5
O7—Mn4—O20	90.9 (2)	H47A—C47—H47C	109.5
O6—Mn4—O20	88.3 (2)	H47B—C47—H47C	109.5
O5—Mn4—O20	86.3 (2)	N15—C48—N14	110.0 (7)
N3—Mn4—O20	88.9 (2)	N15—C48—H48	125.0
O22—Mn4—O20	177.3 (10)	N14—C48—H48	125.0
O7—Mn4—N10	91.7 (6)	C50—C49—N14	107.9 (7)
O6—Mn4—N10	89.0 (6)	C50—C49—H49	126.1
O5—Mn4—N10	88.7 (7)	N14—C49—H49	126.1
N3—Mn4—N10	95.7 (7)	C49—C50—N15	107.5 (7)
O20—Mn4—N10	174.6 (6)	C49—C50—H50	126.3
O10—Mn5—O9	177.8 (2)	N15—C50—H50	126.3
O10—Mn5—O8	95.9 (2)	N15—C51—H51A	109.5
O9—Mn5—O8	82.5 (2)	N15—C51—H51B	109.5
O10—Mn5—N4	89.4 (2)	H51A—C51—H51B	109.5
O9—Mn5—N4	92.2 (2)	N15—C51—H51C	109.5
O8—Mn5—N4	174.5 (3)	H51A—C51—H51C	109.5
O10—Mn5—O19	91.3 (2)	H51B—C51—H51C	109.5
O9—Mn5—O19	87.2 (2)	N6—C52—N7	110.9 (7)
O8—Mn5—O19	84.4 (2)	N6—C52—H52	124.6
N4—Mn5—O19	93.7 (2)	N7—C52—H52	124.6
O10—Mn5—N12	91.3 (2)	C54—C53—N6	109.4 (8)
O9—Mn5—N12	90.1 (2)	C54—C53—H53	125.3
O8—Mn5—N12	91.3 (2)	N6—C53—H53	125.3
N4—Mn5—N12	90.3 (2)	C53—C54—N7	106.9 (7)
O19—Mn5—N12	175.2 (2)	C53—C54—H54	126.6
O13—Mn6—O12	174.6 (2)	N7—C54—H54	126.6
O13—Mn6—N5	87.7 (2)	N7—C55—H55A	109.5
O12—Mn6—N5	93.5 (2)	N7—C55—H55B	109.5
O13—Mn6—N14	87.6 (2)	H55A—C55—H55B	109.5
O12—Mn6—N14	90.5 (2)	N7—C55—H55C	109.5
N5—Mn6—N14	171.0 (3)	H55A—C55—H55C	109.5
O13—Mn6—O11	107.3 (2)	H55B—C55—H55C	109.5
O12—Mn6—O11	77.9 (2)	O17—C56—O16	123.0 (7)
N5—Mn6—O11	93.7 (2)	O17—C56—C57	120.2 (7)
N14—Mn6—O11	95.0 (2)	O16—C56—C57	116.8 (7)
O13—Mn6—O16	107.0 (2)	C56—C57—H57A	109.5
O12—Mn6—O16	68.0 (2)	C56—C57—H57B	109.5
N5—Mn6—O16	83.3 (2)	H57A—C57—H57B	109.5
N14—Mn6—O16	90.7 (2)	C56—C57—H57C	109.5
O11—Mn6—O16	145.4 (2)	H57A—C57—H57C	109.5
C1—O1—Mn2	131.9 (5)	H57B—C57—H57C	109.5
C7—O2—Mn3	110.3 (5)	O19—C58—O18	126.0 (7)
N1—O3—Mn3	115.7 (4)	O19—C58—C59	117.7 (8)
N1—O3—Mn1	112.8 (4)	O18—C58—C59	116.2 (8)
Mn3—O3—Mn1	119.2 (2)	C58—C59—H59A	109.5
C8—O4—Mn3	129.1 (5)	C58—C59—H59B	109.5
C14—O5—Mn4	111.9 (5)	H59A—C59—H59B	109.5
N2—O6—Mn4	112.7 (4)	C58—C59—H59C	109.5
N2—O6—Mn1	124.0 (4)	H59A—C59—H59C	109.5
Mn4—O6—Mn1	123.2 (3)	H59B—C59—H59C	109.5
C15—O7—Mn4	124.9 (5)	O21—C63—N17	126.0 (11)
C21—O8—Mn5	111.0 (5)	O21—C63—H63	117.0
N3—O9—Mn5	110.8 (4)	N17—C63—H63	117.0
N3—O9—Mn1	117.3 (4)	N17—C64—H64A	109.5
Mn5—O9—Mn1	112.4 (2)	N17—C64—H64B	109.5
C22—O10—Mn5	130.5 (5)	H64A—C64—H64B	109.5
C28—O11—Mn6	110.2 (5)	N17—C64—H64C	109.5
N4—O12—Mn6	117.1 (4)	H64A—C64—H64C	109.5
N4—O12—Mn1	114.2 (4)	H64B—C64—H64C	109.5
Mn6—O12—Mn1	107.3 (2)	N17—C65—H65A	109.5
C29—O13—Mn6	132.4 (5)	N17—C65—H65B	109.5
C35—O14—Mn2	109.4 (5)	H65A—C65—H65B	109.5
N5—O15—Mn2	118.1 (4)	N17—C65—H65C	109.5
N5—O15—Mn1	112.2 (4)	H65A—C65—H65C	109.5
Mn2—O15—Mn1	109.8 (2)	H65B—C65—H65C	109.5
C56—O16—Mn1	130.8 (5)	C60—O20—Mn4	123.1 (5)
C56—O16—Mn6	133.1 (5)	O20—C60—N16	124.5 (8)
Mn1—O16—Mn6	92.22 (19)	O20—C60—H60	117.8
C56—O17—Mn3	128.8 (4)	N16—C60—H60	117.8
C58—O18—Mn1	128.1 (5)	C60—N16—C62	123.0 (7)
C58—O19—Mn5	135.8 (5)	C60—N16—C61	119.9 (7)
C7—N1—O3	113.8 (6)	C62—N16—C61	117.1 (7)
C7—N1—Mn2	132.3 (5)	N16—C61—H61A	109.5
O3—N1—Mn2	113.7 (4)	N16—C61—H61B	109.5
C14—N2—O6	112.2 (6)	H61A—C61—H61B	109.5
C14—N2—Mn3	130.6 (6)	N16—C61—H61C	109.5
O6—N2—Mn3	117.2 (4)	H61A—C61—H61C	109.5
C21—N3—O9	112.5 (6)	H61B—C61—H61C	109.5
C21—N3—Mn4	128.7 (5)	N16—C62—H62A	109.5
O9—N3—Mn4	118.5 (4)	N16—C62—H62B	109.5
C28—N4—O12	113.7 (6)	H62A—C62—H62B	109.5
C28—N4—Mn5	134.2 (6)	N16—C62—H62C	109.5
O12—N4—Mn5	112.1 (4)	H62A—C62—H62C	109.5
C35—N5—O15	112.7 (6)	H62B—C62—H62C	109.5
C35—N5—Mn6	134.0 (5)	C40—N10—C41	105.9 (19)
O15—N5—Mn6	113.1 (4)	C40—N10—Mn4	126.8 (19)
C52—N6—C53	105.7 (7)	C41—N10—Mn4	127.2 (14)
C52—N6—Mn2	126.9 (5)	N10—C40—N11	110.3 (18)
C53—N6—Mn2	127.3 (6)	N10—C40—H40	124.8
C52—N7—C54	107.1 (7)	N11—C40—H40	124.8
C52—N7—C55	126.0 (7)	C42—C41—N10	110.4 (17)
C54—N7—C55	126.9 (7)	C42—C41—H41	124.8
C36—N8—C37	104.5 (7)	N10—C41—H41	124.8
C36—N8—Mn3	126.1 (6)	C41—C42—N11	104.3 (17)
C37—N8—Mn3	127.8 (6)	C41—C42—H42	127.9
C36—N9—C38	107.4 (8)	N11—C42—H42	127.9
C36—N9—C39	126.4 (8)	C40—N11—C42	108.7 (15)
C38—N9—C39	126.2 (8)	C40—N11—C43	125.0 (18)
C44—N12—C45	104.2 (7)	C42—N11—C43	125.7 (19)
C44—N12—Mn5	125.3 (5)	N11—C43—H43A	109.5
C45—N12—Mn5	129.5 (6)	N11—C43—H43B	109.5
C44—N13—C46	107.8 (8)	H43A—C43—H43B	109.5
C44—N13—C47	125.3 (8)	N11—C43—H43C	109.5
C46—N13—C47	126.9 (8)	H43A—C43—H43C	109.5
C48—N14—C49	106.2 (6)	H43B—C43—H43C	109.5
C48—N14—Mn6	126.6 (5)	C40B—O22—Mn4	130 (3)
C49—N14—Mn6	127.3 (5)	O22—C40B—N11B	125 (3)
C48—N15—C50	108.4 (7)	O22—C40B—H40B	117.7
C48—N15—C51	124.9 (7)	N11B—C40B—H40B	117.7
C50—N15—C51	126.7 (7)	C40B—N11B—C42B	120 (2)
C63—N17—C65	122.6 (11)	C40B—N11B—C43B	124 (3)
C63—N17—C64	119.2 (10)	C42B—N11B—C43B	116 (3)
C65—N17—C64	118.2 (11)	N11B—C42B—H42A	109.5
O1—C1—C2	116.9 (7)	N11B—C42B—H42B	109.5
O1—C1—C6	124.4 (7)	H42A—C42B—H42B	109.5
C2—C1—C6	118.7 (8)	N11B—C42B—H42C	109.5
C3—C2—C1	121.1 (8)	H42A—C42B—H42C	109.5
C3—C2—H2	119.4	H42B—C42B—H42C	109.5
C1—C2—H2	119.4	N11B—C43B—H43D	109.5
C4—C3—C2	119.4 (8)	N11B—C43B—H43E	109.5
C4—C3—H3	120.3	H43D—C43B—H43E	109.5
C2—C3—H3	120.3	N11B—C43B—H43F	109.5
C5—C4—C3	120.7 (9)	H43D—C43B—H43F	109.5
C5—C4—H4	119.7	H43E—C43B—H43F	109.5
C3—C4—H4	119.7	C68—C67—H67A	109.5
C4—C5—C6	121.0 (8)	C68—C67—H67B	109.5
C4—C5—H5	119.5	H67A—C67—H67B	109.5
C6—C5—H5	119.5	C68—C67—H67C	109.5
C5—C6—C1	119.0 (8)	H67A—C67—H67C	109.5
C5—C6—C7	118.0 (7)	H67B—C67—H67C	109.5
C1—C6—C7	123.0 (8)	O23—C68—C67	111 (2)
O2—C7—N1	120.6 (7)	O23—C68—H68A	109.5
O2—C7—C6	120.6 (7)	C67—C68—H68A	109.5
N1—C7—C6	118.8 (7)	O23—C68—H68B	109.5
O4—C8—C9	116.9 (7)	C67—C68—H68B	109.5
O4—C8—C13	123.8 (7)	H68A—C68—H68B	108.1
C9—C8—C13	119.3 (7)	C68—O23—C69	111.1 (18)
C10—C9—C8	120.1 (8)	O23—C69—C70	106 (2)
C10—C9—H9	119.9	O23—C69—H69A	110.6
C8—C9—H9	119.9	C70—C69—H69A	110.6
C11—C10—C9	120.9 (8)	O23—C69—H69B	110.6
C11—C10—H10	119.5	C70—C69—H69B	110.6
C9—C10—H10	119.5	H69A—C69—H69B	108.8
C12—C11—C10	119.5 (8)	C69—C70—H70A	109.5
C12—C11—H11	120.3	C69—C70—H70B	109.5
C10—C11—H11	120.3	H70A—C70—H70B	109.5
C11—C12—C13	122.1 (8)	C69—C70—H70C	109.5
C11—C12—H12	119.0	H70A—C70—H70C	109.5
C13—C12—H12	119.0	H70B—C70—H70C	109.5
C8—C13—C12	118.1 (8)	C68B—C67B—H67D	109.5
C8—C13—C14	124.2 (7)	C68B—C67B—H67E	109.5
C12—C13—C14	117.7 (8)	H67D—C67B—H67E	109.5
O5—C14—N2	120.8 (7)	C68B—C67B—H67F	109.5
O5—C14—C13	120.2 (7)	H67D—C67B—H67F	109.5
N2—C14—C13	119.0 (7)	H67E—C67B—H67F	109.5
O7—C15—C20	123.0 (7)	O23B—C68B—C67B	112 (2)
O7—C15—C16	117.7 (7)	O23B—C68B—H68C	109.3
C20—C15—C16	119.3 (8)	C67B—C68B—H68C	109.3
C17—C16—C15	119.3 (8)	O23B—C68B—H68D	109.3
C17—C16—H16	120.4	C67B—C68B—H68D	109.3
C15—C16—H16	120.4	H68C—C68B—H68D	107.9
C18—C17—C16	121.0 (8)	C68B—O23B—C69B	113 (3)
C18—C17—H17	119.5	O23B—C69B—C70B	106 (2)
C16—C17—H17	119.5	O23B—C69B—H69C	110.5
C17—C18—C19	120.1 (9)	C70B—C69B—H69C	110.5
C17—C18—H18	119.9	O23B—C69B—H69D	110.5
C19—C18—H18	119.9	C70B—C69B—H69D	110.5
C18—C19—C20	120.6 (8)	H69C—C69B—H69D	108.7
C18—C19—H19	119.7	C69B—C70B—H70D	109.5
C20—C19—H19	119.7	C69B—C70B—H70E	109.5
C15—C20—C19	119.7 (7)	H70D—C70B—H70E	109.5
C15—C20—C21	123.8 (7)	C69B—C70B—H70F	109.5
C19—C20—C21	116.4 (7)	H70D—C70B—H70F	109.5
O8—C21—N3	121.6 (7)	H70E—C70B—H70F	109.5
O8—C21—C20	120.0 (7)	H24A—O24—H24B	120 (10)
N3—C21—C20	118.3 (7)	C72—O25—H25A	109.5
O10—C22—C23	118.0 (8)	O25—C72—H72A	109.5
O10—C22—C27	124.2 (7)	O25—C72—H72B	109.5
C23—C22—C27	117.8 (8)	H72A—C72—H72B	109.5
C24—C23—C22	122.5 (9)	O25—C72—H72C	109.5
C24—C23—H23	118.7	H72A—C72—H72C	109.5
C22—C23—H23	118.7	H72B—C72—H72C	109.5
C23—C24—C25	120.3 (9)	O26—C71—H71A	109.5
C23—C24—H24	119.8	O26—C71—H71B	109.5
C25—C24—H24	119.8	H71A—C71—H71B	109.5
C26—C25—C24	119.3 (8)	O26—C71—H71C	109.5
C26—C25—H25	120.3	H71A—C71—H71C	109.5
C24—C25—H25	120.3	H71B—C71—H71C	109.5
C25—C26—C27	121.3 (9)	C71—O26—H26A	109.5
C25—C26—H26	119.4		
			
N1—Mn2—O1—C1	4.1 (7)	C19—C20—C21—N3	−162.5 (8)
N6—Mn2—O1—C1	−159.4 (7)	Mn5—O10—C22—C23	173.4 (6)
O14—Mn2—O1—C1	99.9 (7)	Mn5—O10—C22—C27	−8.2 (12)
N2—Mn3—O4—C8	25.0 (7)	O10—C22—C23—C24	179.3 (9)
N8—Mn3—O4—C8	−165.4 (7)	C27—C22—C23—C24	0.8 (14)
O2—Mn3—O4—C8	−74.0 (7)	C22—C23—C24—C25	−0.8 (16)
O17—Mn3—O4—C8	112.8 (6)	C23—C24—C25—C26	−0.5 (15)
O5—Mn4—O7—C15	−149.1 (7)	C24—C25—C26—C27	1.7 (14)
N3—Mn4—O7—C15	35.6 (7)	C25—C26—C27—C22	−1.6 (13)
O22—Mn4—O7—C15	−55.9 (13)	C25—C26—C27—C28	179.5 (8)
O20—Mn4—O7—C15	124.5 (7)	O10—C22—C27—C26	−178.1 (8)
N10—Mn4—O7—C15	−60.2 (9)	C23—C22—C27—C26	0.4 (12)
O8—Mn5—O10—C22	−169.4 (7)	O10—C22—C27—C28	0.8 (13)
N4—Mn5—O10—C22	8.8 (7)	C23—C22—C27—C28	179.2 (8)
O19—Mn5—O10—C22	−84.9 (7)	Mn6—O11—C28—N4	0.5 (9)
N12—Mn5—O10—C22	99.1 (7)	Mn6—O11—C28—C27	179.4 (6)
N5—Mn6—O13—C29	5.2 (7)	O12—N4—C28—O11	2.3 (10)
N14—Mn6—O13—C29	177.5 (7)	Mn5—N4—C28—O11	−179.7 (5)
O11—Mn6—O13—C29	−88.0 (7)	O12—N4—C28—C27	−176.6 (6)
O16—Mn6—O13—C29	87.5 (7)	Mn5—N4—C28—C27	1.4 (11)
N1—Mn2—O15—N5	91.7 (5)	C26—C27—C28—O11	2.4 (12)
N6—Mn2—O15—N5	−104.9 (5)	C22—C27—C28—O11	−176.5 (8)
O14—Mn2—O15—N5	−3.4 (4)	C26—C27—C28—N4	−178.7 (7)
N1—Mn2—O15—Mn1	−38.7 (3)	C22—C27—C28—N4	2.4 (12)
N6—Mn2—O15—Mn1	124.7 (3)	Mn6—O13—C29—C30	171.1 (6)
O14—Mn2—O15—Mn1	−133.8 (3)	Mn6—O13—C29—C34	−7.5 (12)
Mn3—O3—N1—C7	7.7 (7)	O13—C29—C30—C31	178.5 (8)
Mn1—O3—N1—C7	−134.4 (5)	C34—C29—C30—C31	−2.8 (13)
Mn3—O3—N1—Mn2	−177.2 (3)	C29—C30—C31—C32	0.7 (14)
Mn1—O3—N1—Mn2	40.6 (5)	C30—C31—C32—C33	0.2 (14)
Mn4—O6—N2—C14	−4.7 (7)	C31—C32—C33—C34	1.2 (14)
Mn1—O6—N2—C14	178.8 (5)	C32—C33—C34—C29	−3.3 (13)
Mn4—O6—N2—Mn3	174.5 (3)	C32—C33—C34—C35	177.4 (8)
Mn1—O6—N2—Mn3	−1.9 (6)	O13—C29—C34—C33	−177.4 (8)
Mn5—O9—N3—C21	−11.7 (7)	C30—C29—C34—C33	4.1 (12)
Mn1—O9—N3—C21	−142.7 (5)	O13—C29—C34—C35	1.9 (12)
Mn5—O9—N3—Mn4	174.0 (3)	C30—C29—C34—C35	−176.6 (7)
Mn1—O9—N3—Mn4	43.0 (6)	Mn2—O14—C35—N5	−4.2 (8)
Mn6—O12—N4—C28	−4.3 (7)	Mn2—O14—C35—C34	177.4 (5)
Mn1—O12—N4—C28	−130.9 (5)	O15—N5—C35—O14	1.7 (10)
Mn6—O12—N4—Mn5	177.2 (3)	Mn6—N5—C35—O14	175.8 (5)
Mn1—O12—N4—Mn5	50.6 (5)	O15—N5—C35—C34	−179.9 (6)
Mn2—O15—N5—C35	2.3 (7)	Mn6—N5—C35—C34	−5.8 (11)
Mn1—O15—N5—C35	131.6 (5)	C33—C34—C35—O14	2.0 (11)
Mn2—O15—N5—Mn6	−173.1 (3)	C29—C34—C35—O14	−177.3 (7)
Mn1—O15—N5—Mn6	−43.8 (5)	C33—C34—C35—N5	−176.4 (7)
Mn2—O1—C1—C2	176.2 (6)	C29—C34—C35—N5	4.3 (11)
Mn2—O1—C1—C6	−3.2 (12)	C37—N8—C36—N9	−1.9 (10)
O1—C1—C2—C3	−178.6 (8)	Mn3—N8—C36—N9	164.7 (6)
C6—C1—C2—C3	0.9 (13)	C38—N9—C36—N8	1.5 (11)
C1—C2—C3—C4	−1.6 (14)	C39—N9—C36—N8	−177.1 (8)
C2—C3—C4—C5	0.2 (14)	C36—N8—C37—C38	1.6 (11)
C3—C4—C5—C6	1.8 (13)	Mn3—N8—C37—C38	−164.7 (7)
C4—C5—C6—C1	−2.4 (12)	N8—C37—C38—N9	−0.7 (11)
C4—C5—C6—C7	176.9 (8)	C36—N9—C38—C37	−0.4 (11)
O1—C1—C6—C5	−179.5 (8)	C39—N9—C38—C37	178.2 (9)
C2—C1—C6—C5	1.0 (12)	C45—N12—C44—N13	1.4 (10)
O1—C1—C6—C7	1.3 (13)	Mn5—N12—C44—N13	−168.2 (5)
C2—C1—C6—C7	−178.2 (8)	C46—N13—C44—N12	−1.1 (11)
Mn3—O2—C7—N1	−6.3 (9)	C47—N13—C44—N12	178.7 (8)
Mn3—O2—C7—C6	174.7 (5)	C44—N12—C45—C46	−1.2 (10)
O3—N1—C7—O2	−0.2 (10)	Mn5—N12—C45—C46	167.7 (6)
Mn2—N1—C7—O2	−174.1 (5)	C44—N13—C46—C45	0.3 (11)
O3—N1—C7—C6	178.8 (6)	C47—N13—C46—C45	−179.6 (9)
Mn2—N1—C7—C6	4.9 (11)	N12—C45—C46—N13	0.6 (11)
C5—C6—C7—O2	−2.3 (11)	C50—N15—C48—N14	−1.8 (10)
C1—C6—C7—O2	176.9 (7)	C51—N15—C48—N14	178.4 (7)
C5—C6—C7—N1	178.7 (7)	C49—N14—C48—N15	2.2 (9)
C1—C6—C7—N1	−2.1 (11)	Mn6—N14—C48—N15	−176.9 (5)
Mn3—O4—C8—C9	157.2 (6)	C48—N14—C49—C50	−1.9 (9)
Mn3—O4—C8—C13	−23.7 (11)	Mn6—N14—C49—C50	177.3 (6)
O4—C8—C9—C10	178.5 (7)	N14—C49—C50—N15	0.8 (10)
C13—C8—C9—C10	−0.7 (11)	C48—N15—C50—C49	0.5 (10)
C8—C9—C10—C11	0.4 (13)	C51—N15—C50—C49	−179.6 (8)
C9—C10—C11—C12	−0.1 (13)	C53—N6—C52—N7	−0.5 (9)
C10—C11—C12—C13	0.1 (12)	Mn2—N6—C52—N7	−177.4 (5)
O4—C8—C13—C12	−178.5 (7)	C54—N7—C52—N6	0.5 (9)
C9—C8—C13—C12	0.7 (11)	C55—N7—C52—N6	−179.1 (9)
O4—C8—C13—C14	2.7 (12)	C52—N6—C53—C54	0.3 (9)
C9—C8—C13—C14	−178.2 (7)	Mn2—N6—C53—C54	177.2 (6)
C11—C12—C13—C8	−0.4 (11)	N6—C53—C54—N7	0.0 (10)
C11—C12—C13—C14	178.5 (7)	C52—N7—C54—C53	−0.3 (9)
Mn4—O5—C14—N2	3.4 (8)	C55—N7—C54—C53	179.3 (9)
Mn4—O5—C14—C13	−178.1 (5)	Mn3—O17—C56—O16	47.4 (11)
O6—N2—C14—O5	0.8 (9)	Mn3—O17—C56—C57	−130.8 (6)
Mn3—N2—C14—O5	−178.2 (5)	Mn1—O16—C56—O17	−6.5 (11)
O6—N2—C14—C13	−177.7 (6)	Mn6—O16—C56—O17	−157.8 (5)
Mn3—N2—C14—C13	3.2 (10)	Mn1—O16—C56—C57	171.8 (5)
C8—C13—C14—O5	−171.6 (7)	Mn6—O16—C56—C57	20.5 (10)
C12—C13—C14—O5	9.5 (10)	Mn5—O19—C58—O18	−20.6 (12)
C8—C13—C14—N2	6.9 (11)	Mn5—O19—C58—C59	158.0 (6)
C12—C13—C14—N2	−171.9 (7)	Mn1—O18—C58—O19	5.9 (11)
Mn4—O7—C15—C20	−32.3 (11)	Mn1—O18—C58—C59	−172.7 (5)
Mn4—O7—C15—C16	148.8 (6)	C65—N17—C63—O21	−178.3 (13)
O7—C15—C16—C17	−179.9 (9)	C64—N17—C63—O21	3.5 (19)
C20—C15—C16—C17	1.1 (14)	Mn4—O20—C60—N16	177.6 (6)
C15—C16—C17—C18	−0.6 (16)	O20—C60—N16—C62	−179.2 (9)
C16—C17—C18—C19	−0.2 (17)	O20—C60—N16—C61	0.0 (13)
C17—C18—C19—C20	0.7 (17)	C41—N10—C40—N11	0 (4)
O7—C15—C20—C19	−179.6 (8)	Mn4—N10—C40—N11	−178 (2)
C16—C15—C20—C19	−0.6 (13)	C40—N10—C41—C42	4 (3)
O7—C15—C20—C21	−0.7 (13)	Mn4—N10—C41—C42	−178.4 (16)
C16—C15—C20—C21	178.2 (8)	N10—C41—C42—N11	−6 (3)
C18—C19—C20—C15	−0.2 (15)	N10—C40—N11—C42	−4 (4)
C18—C19—C20—C21	−179.2 (9)	N10—C40—N11—C43	−176 (3)
Mn5—O8—C21—N3	5.0 (9)	C41—C42—N11—C40	6 (3)
Mn5—O8—C21—C20	−173.0 (6)	C41—C42—N11—C43	178 (3)
O9—N3—C21—O8	4.6 (10)	Mn4—O22—C40B—N11B	173 (4)
Mn4—N3—C21—O8	178.2 (5)	O22—C40B—N11B—C42B	7 (9)
O9—N3—C21—C20	−177.4 (6)	O22—C40B—N11B—C43B	178 (7)
Mn4—N3—C21—C20	−3.8 (11)	C67—C68—O23—C69	−180 (4)
C15—C20—C21—O8	−163.3 (8)	C68—O23—C69—C70	175 (4)
C19—C20—C21—O8	15.5 (12)	C67B—C68B—O23B—C69B	80 (5)
C15—C20—C21—N3	18.6 (12)	C68B—O23B—C69B—C70B	177 (2)

## References

[bb1] Addison, A. W., Rao, T. N., Reedijk, J., van Rijn, J. & Verschoor, G. G. (1984). *J. Chem. Soc. Dalton Trans.* pp. 1349–1356.

[bb2] Bruker (2012). *APEX2* and *SAINT* Bruker AXS Inc., Madison, Wisconsin, USA.

[bb3] Dendrinou-Samara, C., Alevizopoulou, L., Iordanidis, L., Samaras, E. & Kessissoglou, D. P. (2002). *J. Inorg. Biochem.* **89**, 89–96. 10.1016/s0162-0134(01)00415-9 11931968

[bb4] Dendrinou-Samara, C., Papadopoulos, A. N., Malamatari, D. A., Tarushi, A., Raptopoulou, C. P., Terzis, A., Samaras, E. & Kessissoglou, D. P. (2005). *J. Inorg. Biochem.* **99**, 864–875. 10.1016/j.jinorgbio.2004.12.021 15708808

[bb5] Dendrinou-Samara, C., Psomas, G., Iordanidis, L., Tangoulis, V. & Kessissoglou, D. P. (2001). *Chem. Eur. J.* **7**, 5041–5051. 10.1021/ic991476q 11196899

[bb6] Emerich, B., Smith, M., Zeller, M. & Zaleski, C. M. (2010). *J. Chem. Crystallogr.* **40**, 769–777.

[bb7] Hübschle, C. B., Sheldrick, G. M. & Dittrich, B. (2011). *J. Appl. Cryst.* **44**, 1281–1284. 10.1107/S0021889811043202 PMC3246833 22477785

[bb8] Kessissoglou, D. P., Kampf, J. & Pecoraro, V. L. (1994). *Polyhedron*, **13**, 1379–1391.

[bb9] Lah, M. S. & Pecoraro, V. L. (1989). *J. Am. Chem. Soc.* **111**, 7258–7259.

[bb10] Liu, W. & Thorp, H. H. (1993). *Inorg. Chem.* **32**, 4102–4105.

[bb11] Macrae, C. F., Edgington, P. R., McCabe, P., Pidcock, E., Shields, G. P., Taylor, R., Towler, M. & van de Streek, J. (2006). *J. Appl. Cryst.* **39**, 453–457.

[bb12] Mezei, G., Zaleski, C. M. & Pecoraro, V. L. (2007). *Chem. Rev.* **107**, 4933–5003. 10.1021/cr078200h 17999555

[bb13] Pecoraro, V. L. (1989). *Inorg. Chim. Acta* **155**, 171–173.

[bb14] Sheldrick, G. M. (2008*a*). *Acta Cryst.* A**64**, 112–122. 10.1107/S0108767307043930 18156677

[bb15] Sheldrick, G. M. (2008*b*). *CELL_NOW* University of Göttingen, Germany.

[bb16] Sheldrick, G. M. (2009). *TWINABS* University of Göttingen, Germany.

[bb17] Stiefel, E. I. & Brown, G. F. (1972). *Inorg. Chem.* **11**, 434–436.

[bb18] Tigyer, B. R., Zeller, M. & Zaleski, C. M. (2011). *Acta Cryst.* E**67**, m1041–m1042. 10.1107/S160053681102602X PMC3212131 22090833

[bb19] Tigyer, B. R., Zeller, M. & Zaleski, C. M. (2012). *Acta Cryst.* E**68**, m1521–m1522. 10.1107/S1600536812047228 PMC3588767 23468732

[bb20] Westrip, S. P. (2010). *J. Appl. Cryst.* **43**, 920–925.

